# Outlet-Air Relative-Humidity Feedback for Adaptive Binder Delivery During Pulsed Fluidized-Bed Agglomeration of Soy Protein Isolate

**DOI:** 10.3390/foods15162846

**Published:** 2026-08-14

**Authors:** Prarin Chupawa, Chanat Vipattanaporn, Jatupon Saijantha, Surachet Suanjan, Frederik Ronsse, Jan G. Pieters, Donludee Jaisut, Wasan Duangkhamchan

**Affiliations:** 1 Research Unit of Mechatronics Engineering, Faculty of Engineering, Mahasarakham University, Maha Sarakham 44150, Thailand; prarin.c@msu.ac.th; 2 Research Unit of Smart Process Design and Automation, Mahasarakham University, Maha Sarakham 44150, Thailand; 3 Faculty of Engineering, Mahasarakham University, Maha Sarakham 44150, Thailand; chanat.v@msu.ac.th (C.V.); jatapon.s@msu.ac.th (J.S.); surachet.s@msu.ac.th (S.S.); 4 Department of Green Chemistry and Technology, Faculty of Bioscience Engineering, Ghent University, 9000 Gent, Belgium; frederik.ronsse@ugent.be; 5 Department of Plants and Crops, Faculty of Bioscience Engineering, Ghent University, 9000 Gent, Belgium; jan.pieters@ugent.be; 6 Department of Farm Mechanics, Faculty of Agriculture, Kasetsart University, Bangkok 10900, Thailand; donludee.j@ku.th; 7 Research Unit of Process Design and Automation, Faculty of Engineering, Mahasarakham University, Maha Sarakham 44150, Thailand

**Keywords:** soy protein isolate, pulsed fluidized-bed agglomeration, outlet-air relative humidity, adaptive binder delivery, closed-loop process control, powder reconstitution

## Abstract

Fine soy protein isolate (SPI) powders exhibit poor handling and reconstitution properties, while excessive liquid loading can destabilize fluidized-bed agglomeration. This study evaluated an outlet-air relative humidity (RH)-triggered binder-diversion strategy during pulsed fluidized-bed agglomeration of SPI. Atomization pressure (0.5, 1.0, and 1.5 bar), nominal binder pump setting (3.8, 4.7, and 5.6 mL·min^−1^), and operating mode (continuous spraying or RH-triggered control at a 70% set point) were investigated. Under continuous spraying, outlet RH showed an overall increase throughout the 30 min process. RH-triggered operation maintained outlet RH near the set point after threshold attainment by reducing the spray duty cycle to 0.67–0.96. This corresponded to 102.6–131.0 mL of sprayed water per run, approximately 4–33% less than continuous spraying at the same nominal pump setting. Compared with continuous spraying, RH-triggered operation produced a lower mean final moisture content in all nine conditions, a higher mean process yield in seven conditions, higher *D*_50_ values in all conditions, and a lower mean particle size span in seven conditions. Agglomeration reduced the packing-derived *CI* and *HR* and shortened wetting time relative to raw SPI, whereas the effects of RH-triggered operation on flow indices, wetting time, and dispersibility depended on atomization pressure and effective binder delivery. Because the feedback action changed both cumulative binder addition and spray history, the observed product differences represent the performance of the complete RH-triggered control strategy rather than an independent effect of RH stabilization. The findings demonstrate the feasibility of outlet-RH-based adaptive binder delivery during pulsed fluidized-bed agglomeration of SPI.

## 1. Introduction

Soy protein isolate (SPI) is widely used as a functional ingredient in food formulations because of its high protein content and useful technological properties. However, fine SPI powders commonly exhibit poor flowability, high cohesiveness, dust formation, and slow wetting during reconstitution. Fluidized-bed agglomeration can overcome some of these limitations by combining fine primary particles into larger and more porous granules, thereby improving powder handling and water penetration [1,2].

During fluidized-bed agglomeration, atomized binder droplets wet the particle surfaces, and collisions between wetted particles promote liquid-bridge formation and coalescence. Subsequent drying consolidates these liquid bridges and produces more stable agglomerates [3,4,5]. The resulting granule characteristics depend on the balance among wetting, drying, growth, consolidation, breakage, and attrition. Among the operating variables, moisture conditions within the bed are particularly important because they influence liquid-bridge lifetime, particle adhesion, granule growth, and fluidization stability [4,6,7,8,9]. Insufficient moisture restricts agglomerate formation, whereas excessive moisture can cause particle sticking, uncontrolled enlargement, unstable fluidization, and, in severe cases, bed collapse [5,7,8].

Humidity- and moisture-related approaches in fluidized-bed granulation can be distinguished according to both the process variable considered and the way that variable is used. Schaafsma et al. investigated the influence of bed-internal, or interstitial-gas, relative humidity on liquid distribution, particle mixing, and granule growth while applying binder in short, predetermined pulses [8]. In that approach, however, the pulse sequence was specified in advance rather than adjusted in response to a measured RH signal. Inlet-air RH has also been imposed as an experimental operating condition using a humidification system to establish a fluid-bed granulation design space [10], and its influence has been examined in pulsed spray granulation [5,9]. In these studies, inlet-air RH was an imposed condition or process covariate rather than a feedback measurement regulating binder delivery.

Other studies have addressed bed moisture through either model-based prediction or direct solid-moisture measurement. Hu et al. developed a moisture mass-balance model to predict the bed-moisture trajectory from binder spray rate, binder concentration, airflow rate, inlet-air temperature, and inlet-air dew point [6]. This approach supported operating-parameter selection and process scale-up but did not constitute measurement-triggered control of binder delivery. In contrast, true closed-loop strategies have been demonstrated using direct or spectroscopically estimated granule moisture. Watano et al. used an infrared moisture sensor for feedback control of granule moisture [11], while Fujiwara et al. employed online near-infrared measurement and proportionally adjusted the inlet-air temperature according to the difference between measured and target granule moisture [12]. More recently, Tian et al. combined in-line near-infrared moisture estimation with a self-adjusting fuzzy-PID controller [13]. These strategies therefore differ from predetermined pulsed-liquid-feed methods [4,7,8,9], which introduce fixed wetting and drying intervals without adapting the spray sequence to a real-time moisture or humidity signal.

Outlet-air RH has previously been used mainly as a process-monitoring or drying-endpoint variable. For example, Ehlers et al. terminated drying when the difference between inlet- and outlet-air RH remained sufficiently small for a specified period [9]; outlet RH did not control binder delivery during agglomeration. In contrast, the present system uses outlet-air RH measured at the chamber exhaust as a real-time feedback signal to actuate solenoid valves and divert the binder stream between the spray nozzle and drainage line while the pump remains in operation. It therefore regulates effective binder delivery using an indirect gas-phase signal rather than direct granule-moisture measurement or a predetermined spray sequence. Because outlet RH is influenced by evaporation, air temperature, inlet-air humidity, and airflow, it should not be interpreted as a direct measurement of powder or bed moisture. The contribution of this study is therefore the feasibility evaluation of this specific outlet-RH-triggered diversion configuration during pulsed fluidized-bed agglomeration of SPI, rather than the first application of humidity- or moisture-related control in fluidized-bed granulation.

Accordingly, this study evaluated an outlet-air RH-triggered binder-diversion strategy during pulsed fluidized-bed agglomeration of SPI. The effects of atomization pressure, nominal binder pump setting, and operating mode—continuous spraying or RH-triggered control—were investigated in terms of outlet-RH behavior, effective binder delivery, product recovery, moisture content, particle size distribution, packing properties, flow indices, morphology, and reconstitution properties. It was hypothesized that RH-triggered binder diversion would limit prolonged high-RH exposure and adapt liquid input to the moisture-removal capacity of the process. Because the feedback action changed both cumulative binder addition and the temporal spray pattern, differences between controlled and uncontrolled treatments were interpreted as the response to the complete RH-triggered control strategy rather than as an independent effect of RH stabilization alone.

## 2. Materials and Methods

### 2.1. Raw Materials

Soy protein isolate (SPI), purchased from Shandong Sinoglory Health Food Co., Ltd., Qingdao, China, was used as raw powder, while distilled water was used as a binder. The initial moisture content of the raw SPI was 7.14 ± 0.30% wb.

### 2.2. Pulsed Fluidized-Bed Granulator

**Figure 1:**
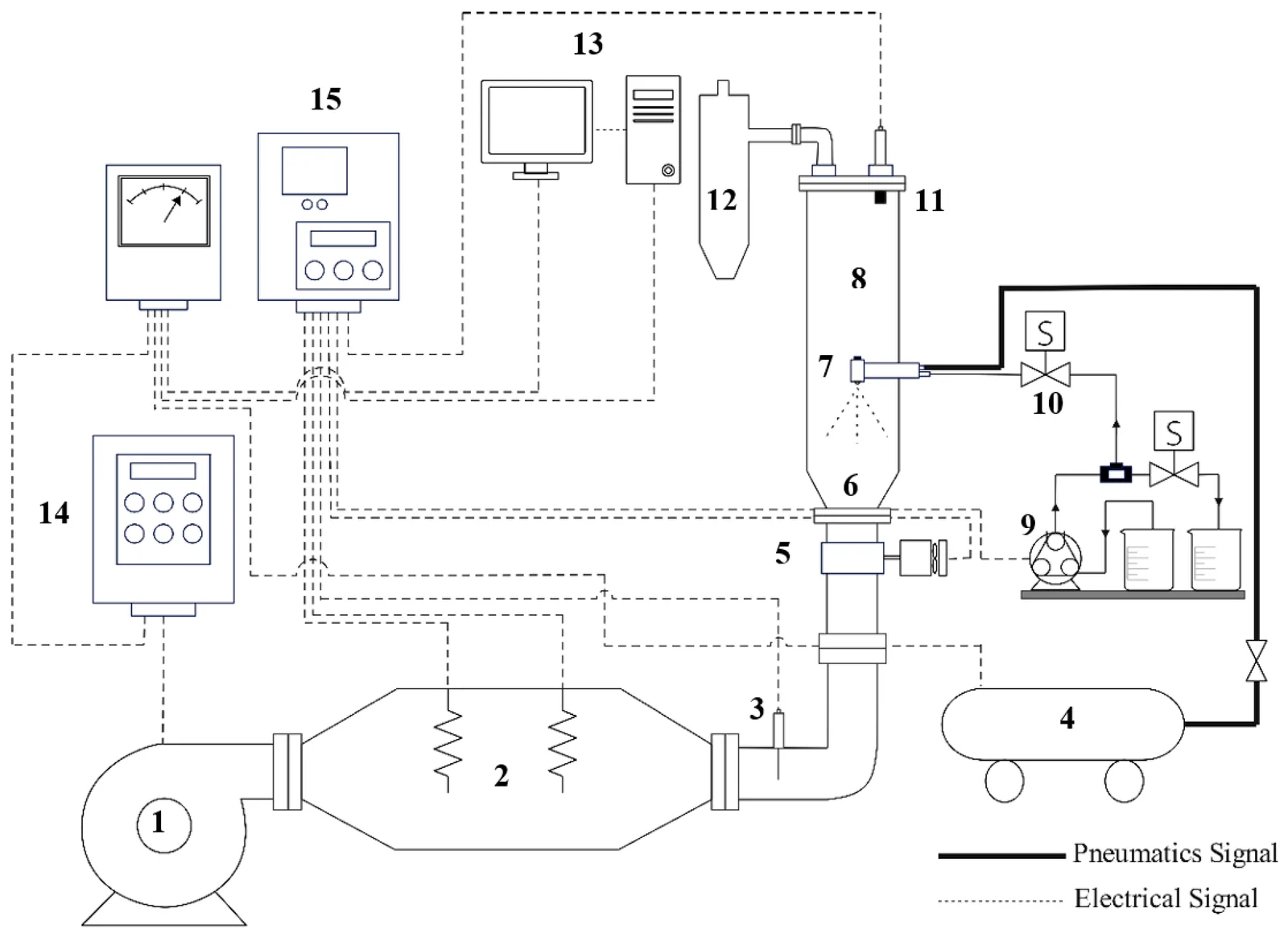
Schematic diagram of the pulsed fluidized-bed granulation system: (1) air blower; (2) air-heating unit; (3) K-type thermocouple; (4) air compressor; (5) butterfly valve with DC motor; (6) wire-mesh air distributor; (7) two-fluid spray nozzle; (8) granulation chamber; (9) peristaltic pump; (10) solenoid valves; (11) outlet-air RH sensor; (12) cyclone separator; (13) computer and data-acquisition system; (14) inverter; and (15) PID temperature controller. Solid and dashed lines represent pneumatic and electrical signals, respectively.

A schematic diagram of the laboratory scale pulsed fluidized-bed agglomeration system is shown in Figure 1. Fluidizing air was supplied by a 1-hp blower driven by an electric motor (Mitsubishi Electric Automation (Thailand) Co., Ltd., Bangkok, Thailand; component 1) and passed through an electric heating unit (component 2). The airflow rate was regulated using an inverter (H-3200 Series, Haitec Transmission Equipment Co., Ltd., Guangzhou, China; component 14). The inlet-air temperature was maintained at 70 °C using a PID controller (MAC-3D, Shimax Co., Ltd., Daisen, Japan; component 15) connected to a K-type thermocouple (component 3).

A 300 g batch of SPI was loaded into a cylindrical polycarbonate chamber with an internal diameter of 0.30 m and a height of 1.0 m (component 8). A wire-mesh air distributor (component 6) supported the powder and prevented particles from entering the air plenum. The superficial air velocity was maintained at 0.50 m·s^−1^. Before binder spraying, the powder bed was fluidized and preheated for 5 min. Each experimental run lasted 30 min. During uncontrolled operation, the binder was sprayed continuously throughout the entire run. During RH-controlled operation, the peristaltic pump operated throughout the 30 min period, while the binder stream was intermittently diverted from the nozzle according to the feedback-control logic. No separate post-spray drying stage was applied.

Fluidizing-air pulsation was generated using a butterfly valve driven by a DC motor and rotational speed controller (component 5). The valve was operated at a frequency of 0.25 Hz using an L298 N motor-driver module controlled by a Raspberry Pi 4B. Each 4 s pulsation cycle comprised 2 s in the open-flow state and 2 s in the closed-flow state, corresponding to a duty cycle of 50%. These operating conditions were maintained throughout all experiments.

Distilled water was supplied as the binder using a peristaltic pump (component 9) and atomized through a two-fluid nozzle (component 7). The nozzle had an orifice diameter of 0.8 mm and was positioned 30 cm above the air distributor in a top spray configuration. Atomizing air was supplied by an air compressor (component 4), and the atomization pressure was adjusted to gauge pressures of 0.5, 1.0, or 1.5 bar. The nominal binder pump settings were 3.8, 4.7, and 5.6 mL·min^−1^.

For RH-controlled operation, two solenoid valves (component 10) redirected the liquid stream either to the spray nozzle or to a drainage line. The control logic is described in Section 2.3. Outlet-air relative humidity was monitored using an SHT31 humidity sensor module (SKU SEN0385, DFRobot, Shanghai, China; component 11) installed at the chamber outlet. Elutriated powder was collected using a cyclone separator (component 12). Component 13 was a computer used to monitor and record the data.

### 2.3. Outlet-Air Relative-Humidity Feedback-Control System

Outlet-air relative humidity (RH) was measured using a capacitive humidity sensor (SHT31, SKU SEN0385, DFRobot, Shanghai, China) installed at the outlet of the fluidized-bed chamber. The sensor signal was acquired by an Arduino Mega 2560 R3 microcontroller (Arduino S.r.l., Monza, Italy) at a sampling interval of 1 s. The stated accuracy of the sensor was ±2% RH within the operating range used in this study. The reported RH values represent measured sensor outputs, and differences within the stated ±2% RH accuracy were not interpreted as physically meaningful. The measured RH and valve operating states were recorded throughout each 30 min experimental run.

A measured outlet-air RH of 70% was selected as an engineering demonstration set point based on the initial continuous spray characterization conducted under the same combinations of atomization pressure and nominal binder pump setting. All continuous spray conditions crossed 70% RH within the 30 min process, allowing controller activation and sufficient time to observe binder diversion and resumption. This value was not derived from the literature as a critical overwetting threshold and was not optimized for SPI agglomeration. Outlet-air RH was treated as an indirect indicator of the gas-phase humidity condition rather than a direct measurement of powder or bed moisture. Evaluation of additional set points is therefore required to determine an appropriate operating range.

The controller operated using an on–off control strategy. The peristaltic pump operated continuously at a nominal binder flow rate, *F_nom_*, of 3.8, 4.7, or 5.6 mL·min^−1^ throughout the 30 min process. When the measured outlet RH was at or below 70%, the solenoid valves directed the distilled-water binder to the two-fluid nozzle. When the RH exceeded 70%, the microcontroller activated the relay modules and switched the solenoid valves to divert the binder to a drainage line, thereby interrupting binder delivery to the bed. Spraying resumed when the RH decreased below the set point.

Because the binder stream was intermittently diverted during RH-controlled operation, the nominal pump setting did not represent the average binder flow delivered to the bed. The spray duty cycle, ∅, was calculated as:

(1) $$\emptyset =\frac{t_{on}}{t_{total}}$$

where *t_on_* is the cumulative time during which binder was delivered to the nozzle and *t_total_* is the total process time of 30 min. The effective average binder flow rate, *F_eff_*, was calculated as:

(2) $$F_{eff}=\emptyset \times F_{nom}$$

The cumulative binder volume delivered to the bed during each run, *V_sprayed_*, was calculated as:

(3) $$V_{sprayed}=F_{nom}t_{on}=F_{eff}t_{total}$$

where *F_nom_* and *F_eff_* are expressed in mL·min^−1^, *t_on_* and *t_total_* are expressed in minutes, and *V_sprayed_* is expressed in mL·run^−1^. During uncontrolled operation, binder was delivered continuously, corresponding to ∅ = 1.

To quantify the outlet-RH dynamics, the recorded RH–time profiles were analyzed using several descriptive indicators. The initial RH (*RH*_0_) was calculated as the mean RH during the first 30 s of the run, and the final RH (*RH_f_*) was calculated as the mean RH during the final 60 s. RH increase rates were estimated by linear regression over selected time intervals and expressed as %RH·s^−1^. The early-stage rate (*r*_0–300_), main humidification rate (*r*_300–900_), and late-stage drift rate (*r*_1200–1800_) were calculated over 0–300, 300–900, and 1200–1800 s, respectively. The time required to reach the RH set point (*t*_70_) was defined as the first time at which the 30 s moving-average RH reached 70%. The cumulative time above 70% RH (*T*_>70_) and integrated RH excess above 70% (*AUC*_>70_) were used to quantify the duration and magnitude of excursions above the selected set point.

**Figure 2:**
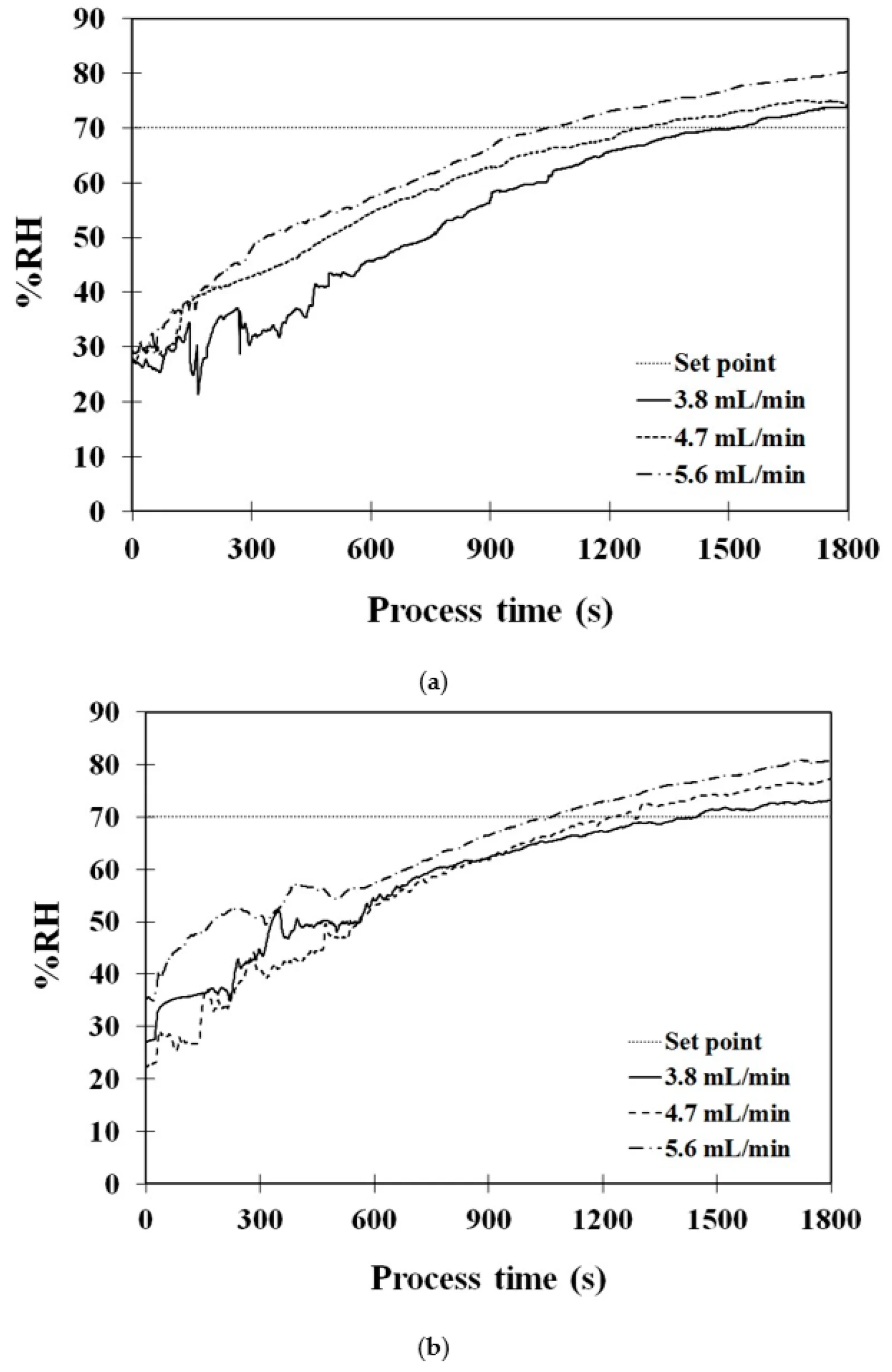

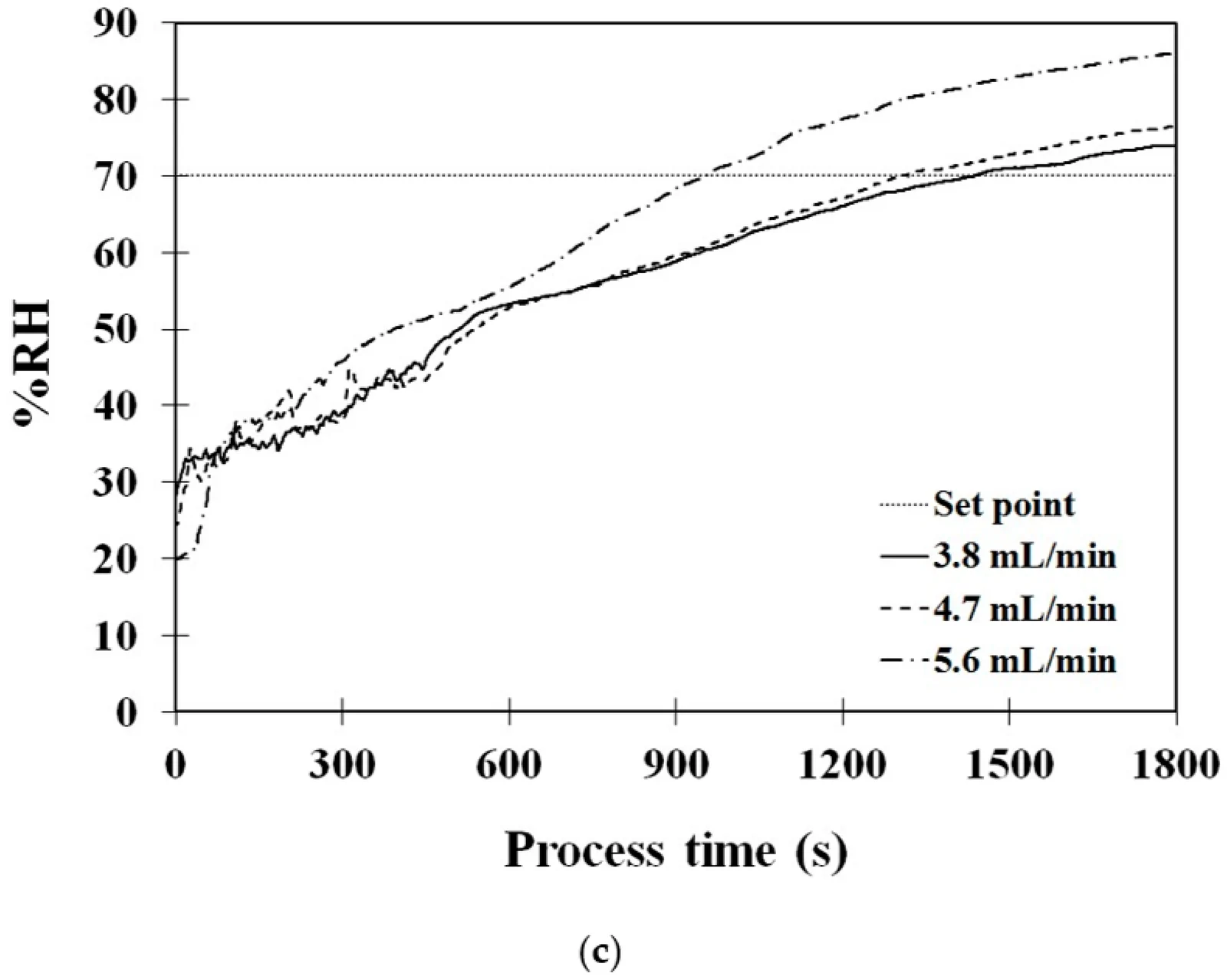
Relative humidity (RH) of the outlet fluidizing air as a function of process time at various nominal binder pump settings (*F_nom_*) and at different atomization pressures: (**a**) 0.5 bar, (**b**) 1.0 bar, and (**c**) 1.5 bar, under uncontrolled agglomeration (without RH control; continuous spraying). The horizontal line indicates the measured controller set point of 70% RH. Quantitative RH descriptors derived from these profiles are provided in Table S1.

**Figure 3:**
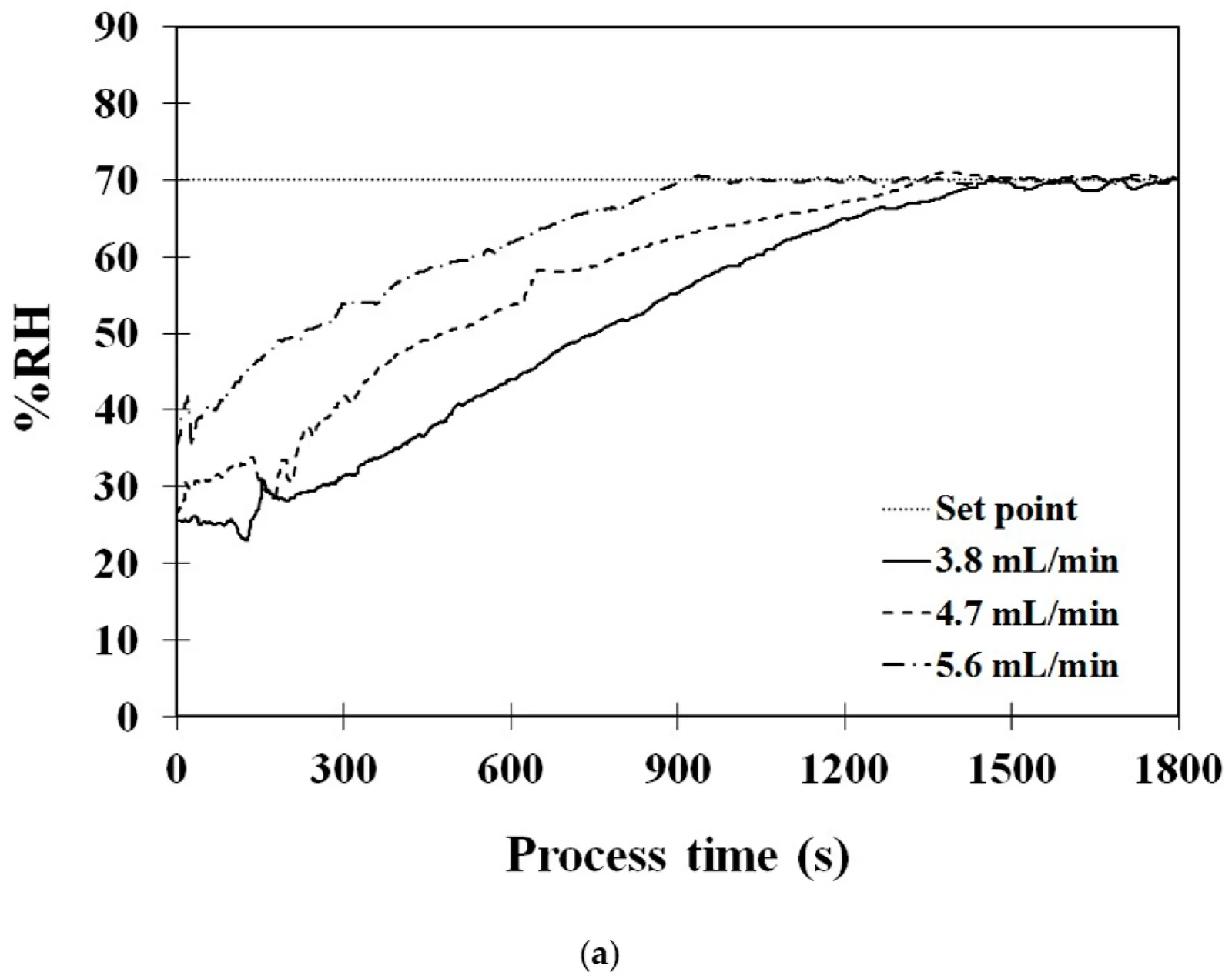

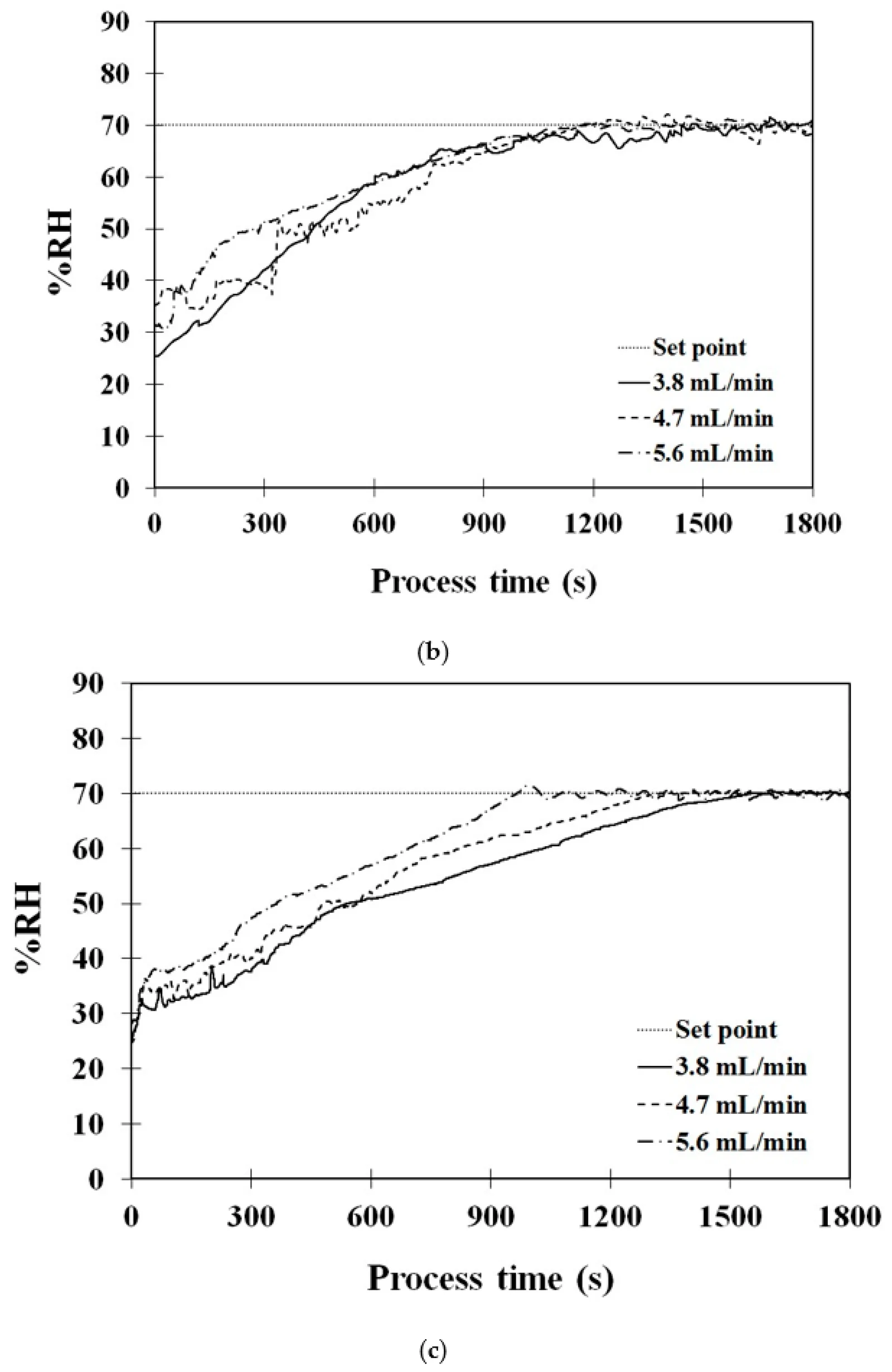
Outlet-air relative humidity (RH) as a function of process time during RH-controlled agglomeration at nominal binder pump settings of 3.8, 4.7, and 5.6 mL·min^−1^ and atomization pressures of (**a**) 0.5 bar, (**b**) 1.0 bar, and (**c**) 1.5 bar. The horizontal line indicates the measured controller set point of 70% RH. During controlled operation, the effective binder delivery differed from the nominal pump setting because the binder stream was intermittently diverted when RH exceeded the set point. Quantitative RH-control descriptors derived from these profiles are provided in Table S1.

Figure 2 and Figure 3 present one experimental trajectory for each operating condition to preserve the actual RH dynamics, because pointwise averaging of duplicate runs could obscure differences in the timing of controller switching. Table S1 summarizes the descriptors calculated from the displayed trajectories in both figures, whereas the binder-delivery parameters in Table 1 were calculated only from the RH-controlled trajectories in Figure 3. Therefore, Table 1 and Table S1 contain descriptive single-run values, while Table 2, Table 3, Table 4, Table 5 and Table 6 report mean ± standard deviation from two independent runs.

### 2.4. Powder and Granule Characterization

#### 2.4.1. Analysis of Moisture Content and Process Yield

The moisture content of the raw and agglomerated SPI samples was determined using the oven-drying method described by Horwitz [14]. Approximately 3 g of each sample was weighed and dried in a hot-air oven at 105 °C for 48 h. Moisture content was expressed on a wet basis (% wb). Moisture content was measured in three analytical replicates for each independent agglomeration run.

At the end of each experimental run, the product remaining in the granulation chamber was collected and passed through an 850 µm sieve. The fraction passing through the sieve was used to determine process yield, whereas the retained fraction was classified as oversized material. For this laboratory-scale study, process yield was operationally defined on a wet-mass basis as the mass of recovered chamber product passing through the 850 µm sieve relative to the initial mass of SPI introduced into the chamber:

(4) $$\mathrm{yield}=\frac{m_{f}}{m_{i}}\times 100$$

where *m_f_* is the wet mass of recovered chamber product passing through the 850 µm sieve and mi is the initial mass of SPI. Material collected in the cyclone, retained above 850 µm, deposited on equipment surfaces, or otherwise not recovered in this chamber fraction was excluded. Because these fractions were not quantified separately, the reported process yield does not represent total material recovery or a complete material balance. Moreover, because it was calculated using wet mass, the value may be influenced by the final product moisture content.

#### 2.4.2. Densities and Apparent Tapped-Bed Porosity

Bulk density (*ρ_b_*) was determined by gently transferring a known mass of powder into a graduated cylinder and recording the occupied volume without compaction. Apparent tapped density after 50 manual taps (*ρ_t,_*_50_) was subsequently determined by manually tapping the cylinder 50 times, following the fixed-tap procedure described by Dacanal et al. [15]. Comparable fixed-number manual-tapping procedures have also been applied in food-powder and food-granule characterization [16,17]. The same fixed-tap procedure was applied to all samples. Because neither an automated tapping device nor a stable-volume endpoint was used, ρ_t,50_ was treated as a comparative packing measurement rather than an equilibrium tapped density. Particle density (*ρ_p_*) was measured using a gas pycnometer (Ultrapyc 1200e, Quantachrome Instruments, Boynton Beach, FL, USA), according to Jinapong et al. [1].

The apparent tapped-bed porosity was calculated using *ρ_t,_*_50_ and *ρ_p_* according to Equation (5). Bulk density, *ρ_t,_*_50_, particle density, and apparent tapped-bed porosity were measured in three analytical replicates for each independent agglomeration run as:

(5) $$\varepsilon_{t}=\left(1-\frac{\rho_{t,\;50}}{\rho_{p}}\right)\times 100$$

where *ρ_t,_*_50_ is the tapped density and *ρ_p_* is the particle density, both expressed in g·mL^−1^.

#### 2.4.3. Measurement of Particle Size Distribution

Particle size distributions of the raw and agglomerated SPI samples were determined by laser diffraction using a particle size analyzer (LA-960V2, HORIBA Ltd., Kyoto, Japan). Measurements were performed using the dry dispersion mode. The particle refractive index was set at 1.53. Each sample was measured in three analytical replicates for each independent agglomeration run.

Particle size distributions were expressed on a volume basis using *D*_10_, *D*_50_, and *D*_90_, corresponding to the particle diameters below which 10%, 50%, and 90% of the cumulative particle volume occurred, respectively. The distribution *span* was calculated as:

(6) $$\mathrm{span}=\frac{D_{90}-D_{10}}{D_{50}}$$

A lower span value indicates a narrower particle size distribution.

#### 2.4.4. Flowability and Cohesiveness Analysis

Carr compressibility index (CI) and Hausner ratio (HR) were calculated from the bulk density (*ρ_b_*) and apparent tapped density after 50 manual taps (*ρ*_*t*,50_), as described in Section 2.4.2. These parameters were used as packing-derived screening indices and not as direct measurements of dynamic powder flow.

(7) $$\mathrm{CI}=\frac{\rho_{t,50}-\rho_{b}}{\rho_{t,50}}\times 100$$

(8) $$\mathrm{HR}=\frac{\rho_{t,50}}{\rho_{b}}$$

For descriptive comparison with previous food-powder studies, *CI* was classified as very good (<15%), good (15–<20%), fair (20–<35%), poor (35–45%), or very poor (>45%), while *HR* was classified as indicating low (<1.20), intermediate (1.20–1.40), or high (>1.40) apparent cohesiveness [15,18]. For each independent agglomeration run, three analytical measurements were averaged before statistical analysis.

#### 2.4.5. Analysis of Reconstitution Properties

Wettability and dispersibility were determined following Jinapong et al. [1]. For wettability, 0.1 g of powder was placed on the surface of 100 mL of distilled water in a 250 mL beaker. The time required for all powder particles to become completely wetted and penetrate the water surface was recorded as the wetting time.

For dispersibility, 10 mL of distilled water was transferred into a 50 mL beaker, and 1 g of powder was added. The mixture was stirred vigorously with a spoon for 15 s using 25 complete back-and-forth movements across the diameter of the beaker. The resulting dispersion was passed through a 212 µm sieve. A 1 mL aliquot of the sieved dispersion was transferred to a pre-weighed aluminum pan and dried at 105 °C for 4 h to determine its total solids content. Dispersibility was calculated as:

(9) $$\mathrm{Dispersibility}\%(\%)=\frac{\left(10+a\right)\times \%TS}{a\times \left(\frac{100-b}{100}\right)}$$

where *a* is the mass of powder used (g), *b* is the moisture content of the powder (% wet basis), and *%TS* is the total solids content (%) of the sieved dispersion. Wettability and dispersibility were measured in three analytical replicates for each independent agglomeration run. Accordingly, dispersibility in this study represents an operational measure of the powder solids passing through a 212 µm sieve after the standardized 15 s mixing procedure; it is not a direct measurement of protein solubility.

#### 2.4.6. Morphology

The raw SPI powder and agglomerated samples were coated with gold before morphological examination using a tabletop scanning electron microscope (TM4000Plus, Hitachi High-Tech Corporation, Tokyo, Japan). The microscope was operated at an accelerating voltage of 15 kV. Images of the raw SPI and agglomerated samples were acquired at ×100 magnification. An additional image of the raw SPI was acquired at ×500 magnification to provide a more detailed view of the primary particles. The SEM micrographs were used only for qualitative morphological observation; no quantitative image analysis was performed.

### 2.5. Experimental Design and Statistical Analysis

A 3 × 3 × 2 factorial arrangement was used to define the treatment combinations, consisting of three atomization pressures (0.5, 1.0, and 1.5 bar), three nominal binder pump settings (3.8, 4.7, and 5.6 mL·min^−1^), and two operating modes: continuous spraying without RH control and RH-triggered spraying at a 70% set point. Each treatment combination was performed in duplicate using two independent agglomeration runs.

For each independent agglomeration run, quantitative powder and granule properties were measured in three analytical replicates unless otherwise stated. The analytical replicate values were averaged to obtain one run-level value for each response variable. For each treatment, the mean and standard deviation were calculated from the two independent run-level values. Therefore, the experimental replication for each treatment was *n* = 2 independent agglomeration runs.

Differences among treatments were assessed using one-way analysis of variance (ANOVA), followed by Duncan’s multiple-range test at a significance level of *p* < 0.05. In this analysis, each combination of atomization pressure, nominal binder pump setting, and operating mode was treated as one treatment group. Raw SPI was included as a reference group for powder-property comparisons when applicable but was not included in process-yield comparisons. Statistical analyses were conducted using IBM SPSS Statistics, version 26 (IBM Corp., Armonk, NY, USA). Results are reported as mean ± standard deviation of two independent agglomeration runs.

## 3. Results and Discussion

### 3.1. Outlet-Air Relative Humidity Under Uncontrolled Operation

Figure 2 shows the outlet-air relative humidity (RH) profiles during uncontrolled agglomeration with continuous binder spraying. Across the tested conditions, outlet RH generally increased throughout the 30 min process. The profiles typically showed an initial increase, followed by a slower rise with local fluctuations. Although some profiles approached a near-constant value toward the end of the process, a consistent steady state RH was not demonstrated across all operating conditions.

The progressive increase in outlet RH demonstrates that the exhaust air became increasingly humid during continuous binder spraying. During agglomeration, part of the atomized water is expected to evaporate and be transported from the chamber, while another fraction may remain temporarily on particle surfaces and within liquid bridges [2,3,19]. However, the outlet-RH signal does not quantify the partitioning of water between the gas and particle phases.

At a given air temperature, higher outlet RH would be consistent with a lower remaining gas-phase drying potential and could increase liquid-bridge lifetime and liquid retention [6,8,20]. Nevertheless, outlet RH is temperature-dependent, and neither outlet-air temperature nor real-time powder moisture was measured. Consequently, the RH profiles cannot distinguish gas-phase humidification from liquid accumulation within the powder bed.

The nominal binder pump setting had a clear influence on RH evolution, as quantified in Table S1. Under continuous spray operation, final RH increased with *F_nom_* at each atomization pressure. At 0.5 bar, RH_f_ increased from 73.8% at 3.8 mL·min^−1^ to 79.9% at 5.6 mL·min^−1^. Similar increases were observed at 1.0 bar, from 72.9 to 80.6%, and at 1.5 bar, from 73.8 to 85.8%. The time required to reach 70% RH also decreased as *F_nom_* increased. For example, at 1.5 bar, *t*_70_ decreased from 1435 s at 3.8 mL·min^−1^ to 952 s at 5.6 mL·min^−1^. These values confirm that higher nominal liquid input accelerated outlet-air humidification and increased the likelihood of high-RH exposure. Although higher atomization pressure can produce finer droplets and promote evaporation, its overall influence also depends on droplet deposition, bed cooling, liquid retention, and the initial humidity condition [4,9,20,21,22]. Therefore, the present results do not demonstrate that increasing atomization pressure always moderated the RH increase.

The duration and integrated magnitude of RH excursions above the selected 70% set point also increased with nominal binder input. Under continuous spray operation, *T*_>70_ ranged from 4.77 to 14.15 min, while *AUC*_>70_ ranged from 11.1 to 141.4%RH·min (Table S1). The highest value occurred at 1.5 bar and 5.6 mL·min^−1^, where outlet RH reached a final value of 85.8% and *AUC*_>70_ increased to 141.4%RH·min. The late-stage RH change rate, *r*_1200–1800_, remained positive in all uncontrolled runs, ranging from 0.010 to 0.015%RH·s^−1^. This indicates that outlet RH was still increasing slowly near the end of the 30 min process rather than reaching a consistent steady state.

At a given air temperature, increasingly humid exhaust air would generally correspond to a smaller vapor-pressure gradient between wetted particle surfaces and the surrounding air. Under continuous binder addition, this condition could prolong liquid-bridge lifetimes and increase the likelihood of liquid retention, overwetting, excessive granule growth, wall deposition, or unstable fluidization [5,6,7,8]. These are possible mechanisms based on established fluidized-bed behavior rather than directly observed responses. Bed moisture, pressure drop, wall deposition, and fluidization stability were not measured; therefore, the occurrence or severity of these phenomena cannot be determined from the outlet-RH profiles.

### 3.2. Outlet-Air Relative Humidity Under Rh-Triggered Feedback Control

Figure 3 shows the outlet-air relative humidity (RH) profiles during agglomeration with RH-triggered binder diversion. Under all operating conditions, outlet RH initially increased while the binder was directed to the spray nozzle. When the measured RH exceeded the 70% set point, the controller diverted the binder away from the nozzle. Binder spraying resumed when RH decreased below the set point. Consequently, the profiles showed two general stages: an initial increase toward the set point, followed by a regulated stage in which RH fluctuated around 70%. Unlike uncontrolled operation, no continued upward RH drift was observed during the later stage of the controlled runs.

The small fluctuations observed after the set point was reached were consistent with the on–off operating principle of the controller, which repeatedly interrupted and resumed binder delivery. Therefore, this stage should be described as a regulated outlet-RH regime, rather than as a quasi steady powder-moisture state. Outlet RH represents the humidity condition of the exhaust air and is influenced by air temperature; it does not directly measure the instantaneous moisture content of the powder bed [6,8,20].

The time required to reach the 70% set point, *t*_70_, ranged from 966 to 1552 s under RH-controlled operation (Table S1). At 1.0 and 1.5 bar, increasing *F_nom_* generally shortened *t*_70_. For example, at 1.5 bar, *t*_70_ decreased from 1552 s at 3.8 mL·min^−1^ to 966 s at 5.6 mL·min^−1^. At 0.5 bar, *t*_70_ decreased from 1469 s at 3.8 mL·min^−1^ to 1338–1357 s at the higher pump settings. However, because the initial RH differed among profiles, the time to reach 70% RH should be interpreted together with RH_0_, not as a result of binder input alone.

For the displayed single-run trajectories, the duration (*T*_>70_) and integrated magnitude (*AUC*_>70_) of excursions above the measured set point were lower under RH-triggered operation than under continuous spraying. *T*_>70_ ranged from 4.8 to 14.2 min under continuous spraying and from 1.1 to 6.6 min under RH-triggered operation, while *AUC*_>70_ ranged from 11.1 to 141.4 and from 0.2 to 4.5%RH·min, respectively (Table S1). These quantities were calculated relative to the measured 70% RH signal and should be interpreted as descriptive sensor-based metrics rather than precise exposure above an absolute true-RH threshold. Final controlled readings were approximately 69–70%, and differences within this range were not interpreted because they were smaller than the stated ±2% RH sensor accuracy. The broader observation is that the displayed controlled profiles remained near the measured set point, whereas the continuous spray profiles continued to increase and ended at approximately 73–86% RH.

The feedback strategy limited the duration and magnitude of measured outlet-RH excursions above the selected set point. At a given air temperature, this may help avoid prolonged exposure to increasingly humid exhaust-air conditions [6,8,20]. However, the results do not establish 70% RH as the onset of overwetting, wall deposition, excessive granule growth, or bed instability, nor do they demonstrate that the controller prevented these phenomena. Because bed moisture, pressure drop, wall deposition, and fluidization stability were not measured, the demonstrated control outcome is restricted to regulation of the measured outlet-air RH.

The feedback action also changed the quantity and temporal pattern of binder delivered to the bed. The spray duty cycle, cumulative spray-on time, effective binder flow rate, and cumulative sprayed-water volume under each operating condition are summarized in Table 1.

**Table 1:** Spray duty cycle and effective binder-delivery parameters during RH-controlled agglomeration at an outlet-RH set point of 70%.

P (bar)	*F_nom_* (mL·min^−1^)	∅ (-)	*t_on_* (min)	*F_eff_* (mL·min^−1^)	*V_sprayed_* (mL·run^−1^)
0.5	3.8	0.96	28.8	3.65	109.44
0.5	4.7	0.79	23.7	3.71	111.39
0.5	5.6	0.78	23.4	4.37	131.04
1.0	3.8	0.93	27.9	3.53	106.02
1.0	4.7	0.90	27.0	4.23	126.90
1.0	5.6	0.67	20.1	3.75	112.56
1.5	3.8	0.90	27.0	3.42	102.60
1.5	4.7	0.78	23.4	3.67	109.98
1.5	5.6	0.73	21.9	4.09	122.64

P, atomization pressure; *F_nom_*, nominal binder pump setting; ∅, spray duty cycle; *t_on_*, cumulative spray-on time; *F_eff_*, effective average binder flow rate; and *V_sprayed_*, cumulative sprayed-water volume. Values are descriptive single-run parameters calculated from the RH-controlled trajectories displayed in Figure 3. They are not replicate means; therefore, between-run standard deviations are not reported.

Across the RH-controlled conditions, the spray duty cycle ranged from 0.67 to 0.96, corresponding to cumulative spray-on times of 20.1–28.8 min, effective binder flow rates of 3.42–4.37 mL·min^−1^, and cumulative sprayed-water volumes of 102.6–131.0 mL per run. These duty cycles indicate that binder delivery was interrupted for approximately 4–33% of the total processing time relative to continuous spraying at the same nominal pump setting.

At each atomization pressure, the spray duty cycle decreased as the nominal binder pump setting increased, indicating that higher nominal liquid input resulted in a greater cumulative binder-diversion time to maintain outlet RH near the set point. However, the cumulative sprayed-water volume did not necessarily increase with the nominal pump setting because the reduction in duty cycle could offset the higher pump rate. For example, at 1.0 bar, increasing *F_nom_* from 4.7 to 5.6 mL·min^−1^ reduced the duty cycle from 0.90 to 0.67 and decreased *V_sprayed_* from 126.90 to 112.56 mL per run.

These results demonstrate that the controller adapted the effective liquid input in response to the outlet-RH trajectory. Consequently, comparisons between controlled and uncontrolled treatments should consider the effective binder delivery and spray sequence rather than the nominal pump setting alone.

### 3.3. Moisture Content and Process Yield

Table 2 presents the final moisture content and process yield obtained under continuous spray and RH-controlled operation. Because RH control was implemented by intermittently diverting the binder stream, controlled and uncontrolled treatments at the same nominal pump setting did not receive identical cumulative binder volumes. Therefore, differences between the two operating modes should be interpreted as responses to the complete RH-triggered binder-delivery strategy rather than to RH regulation alone.

**Table 2:** Final moisture content and process yield at different atomization pressures (P) and nominal binder pump settings (F_nom_) under continuous spray and RH-controlled agglomeration.

Condition		Moisture Content (% wb)		Process Yield (%; Wet-Mass Basis)	
P	*F_nom_*	No Control	Control	No Control	Control
0.5	3.8	12.17 ± 0.45 ^abc^	9.08 ± 1.00 ^ghi^	57.33 ± 5.21 ^f^	58.44 ± 2.99 ^f^
0.5	4.7	12.81 ± 0.57 ^a^	9.95 ± 0.16 ^efgh^	60.22 ± 2.27 ^ef^	74.22 ± 3.66 ^a^
0.5	5.6	13.12 ± 0.67 ^a^	11.40 ± 0.52 ^bcd^	48.40 ± 0.81 ^g^	70.33 ± 4.91 ^abc^
1.0	3.8	10.23 ± 0.59 ^defg^	9.10 ± 0.77 ^ghi^	63.08 ± 0.79 ^cdef^	64.79 ± 1.55 ^bcdef^
1.0	4.7	10.99 ± 0.80 ^cde^	9.49 ± 0.27 ^fghi^	67.04 ± 1.06 ^abcde^	68.75 ± 5.57 ^abcd^
1.0	5.6	12.26 ± 0.99 ^ab^	10.71 ± 1.07 ^def^	70.36 ± 0.63 ^abc^	68.71 ± 5.13 ^abcd^
1.5	3.8	8.89 ± 0.31 ^hi^	8.35 ± 0.31 ^i^	49.87 ± 8.08 ^g^	59.29 ± 2.03 ^ef^
1.5	4.7	10.93 ± 0.71 ^cde^	9.98 ± 1.16 ^efgh^	63.67 ± 3.18 ^cdef^	72.64 ± 7.84 ^ab^
1.5	5.6	10.28 ± 0.61 ^defg^	8.64 ± 0.76 ^i^	63.11 ± 5.43 ^cdef^	61.44 ± 1.50 ^def^
Raw		7.14 ± 0.30 ^j^		-	-

Different superscript letters within the same response variable indicate significant differences among treatment groups according to one-way ANOVA followed by Duncan’s multiple-range test (*p* < 0.05). P, atomization pressure; *F_nom_*, nominal binder pump setting. Raw SPI was included only for moisture-content comparison and was not included in process-yield comparison. During RH-controlled operation, effective binder delivery differed from *F_nom_*; the corresponding duty cycle and effective binder-delivery parameters are reported in Table 1. Process yield was defined as the wet-mass percentage of recovered chamber product passing through the 850 µm sieve relative to the initial SPI mass; it does not represent total material recovery.

#### 3.3.1. Final Moisture Content

Under continuous spray operation, the final moisture content ranged from 8.89 to 13.12% wb, whereas values under RH-controlled operation ranged from 8.35 to 11.40% wb (Table 2). The mean moisture content was lower under RH-controlled operation in all nine operating conditions, although the magnitude and statistical significance of the reduction varied among conditions. The largest reductions were observed at 0.5 bar, where moisture content decreased from 12.17 to 9.08% wb at *F_nom_* = 3.8 mL·min^−1^ and from 12.81 to 9.95% wb at *F_nom_* = 4.7 mL·min^−1^.

At 0.5 and 1.0 bar, mean moisture content increased with the nominal pump setting under both operating modes. Under RH-controlled operation, however, this trend did not always correspond directly to cumulative binder volume, indicating that the instantaneous spray rate and temporal spray sequence also influenced final moisture content [7,9,13]. At 1.5 bar, the response was not monotonic, suggesting that the final moisture content was governed by the combined effects of binder delivery, atomization, evaporation, and liquid retention within the bed [6,13,20].

The lower moisture-content means under RH-controlled operation were consistent with the reduced cumulative binder volumes reported in Table 1 and with the spray-off intervals introduced when outlet RH exceeded the set point. These spray-off periods allowed fluidizing air to continue removing moisture while no additional binder was delivered [7,9,13]. However, the respective contributions of the regulated RH trajectory and reduced liquid addition cannot be separated using the present experimental design.

#### 3.3.2. Process Yield

Process yield, operationally defined as the wet-mass recovery of chamber product passing through the 850 µm sieve, ranged from 48.40 to 70.36% under continuous spray operation and from 58.44 to 74.22% under RH-controlled operation (Table 2). A higher mean process yield was observed under RH-controlled operation in seven of the nine conditions, although not all differences between matched conditions were statistically significant. The largest numerical differences occurred at 0.5 bar and nominal pump settings of 4.7 and 5.6 mL·min^−1^. However, this pattern was not universal, as slightly lower yields were obtained under RH-controlled operation at 1.0 and 1.5 bar with the 5.6 mL·min^−1^ setting.

Material excluded from the yield calculation may have included product retained above 850 µm, cyclone fines, deposits on chamber or equipment surfaces, and other unrecovered fractions. Formation of oversized material under high-wetting conditions and elutriation of fine particles at high atomization pressure represent possible loss pathways [2,4,5,6,8,9,23,24]. However, these material streams were not quantified separately, and pressure drop and fluidization stability were not monitored. Consequently, the principal source of unrecovered material cannot be identified, and the yield differences cannot demonstrate that RH-triggered operation prevented overwetting, wall deposition, or unstable fluidization.

Laboratory-scale studies have reported process yields of 55.4–90.5% for pulsed fluidized-bed agglomeration of SPI [25] and dry-basis yields of 46.2–59.6% for fluidized-bed agglomeration of Spirulina powder [24]. These published values provide only general laboratory scale context and should not be treated as direct benchmarks because of differences in equipment, powder properties, binders, yield definitions, moisture basis, and product-collection procedures. The present results are therefore suitable primarily for comparing treatments within this laboratory system and do not establish an acceptable industrial yield. Industrial evaluation would require a complete component-level dry solids mass balance, quantification and potential recovery or recycling of excluded fractions, and assessment under scale-up conditions.

### 3.4. Particle Size Distribution and Morphology

#### 3.4.1. Particle Size Distribution

Particle size distributions of the raw SPI and agglomerated products are summarized in Table 3 using *D*_10_, *D*_50_, *D*_90_, and span. The raw SPI had a median particle diameter (*D*_50_) of 46.32 µm, whereas the agglomerated samples had *D*_50_ values ranging from 142.93 to 384.65 µm. These results confirm substantial particle enlargement during pulsed fluidized-bed agglomeration [2,3,15].

**Table 3:** Particle size distribution of raw SPI and agglomerated products obtained at different atomization pressures (P) and nominal binder pump settings (*F_nom_*) under continuous spray and RH-controlled operation.

Condition		D_10_ (µm)		D_50_ (µm)		D_90_ (µm)		Span (-)	
P	*F_nom_*	No Control	Control	No Control	Control	No Control	Control	No Control	Control
0.5	3.8	141.57 ± 2.79 ^d^	166.76 ± 2.58 ^a^	336.31 ± 7.20 ^d^	384.65 ± 3.94 ^a^	594.88 ± 8.23 ^c^	661.95 ± 8.50 ^ab^	1.35 ± 0.03 ^ef^	1.29 ± 0.01 ^ghi^
0.5	4.7	109.20 ± 2.69 ^fg^	159.26 ± 7.93 ^b^	281.46 ± 5.89 ^g^	359.22 ± 5.43 ^c^	517.55 ± 10.77 ^f^	666.09 ± 14.39 ^a^	1.45 ± 0.00 ^c^	1.41 ± 0.08 ^cd^
0.5	5.6	121.77 ± 3.78 ^e^	147.77 ± 1.56 ^c^	308.48 ± 5.42 ^f^	368.08 ± 2.71 ^b^	564.00 ± 9.65 ^d^	652.02 ± 5.43 ^b^	1.43 ± 0.06 ^c^	1.37 ± 0.02 ^de^
1.0	3.8	97.04 ± 1.64 ^i^	117.90 ± 1.71 ^e^	234.98 ± 1.18 ^k^	284.03 ± 4.20 ^g^	442.52 ± 2.95 ^i^	549.65 ± 4.42 ^e^	1.47 ± 0.01 ^bc^	1.52 ± 0.02 ^ab^
1.0	4.7	92.72 ± 0.52 ^j^	108.10 ± 1.02 ^gh^	206.44 ± 0.68 ^l^	242.78 ± 2.40 ^j^	406.19 ± 4.93 ^k^	453.76 ± 5.91 ^h^	1.52 ± 0.02 ^ab^	1.42 ± 0.02 ^c^
1.0	5.6	112.63 ± 1.12 ^f^	159.60 ± 2.06 ^b^	250.18 ± 3.04 ^i^	327.59 ± 0.99 ^e^	453.07 ± 1.37 ^h^	564.24 ± 0.43 ^d^	1.36 ± 0.02 ^def^	1.24 ± 0.01 ^i^
1.5	3.8	68.11 ± 0.66 ^l^	70.55 ± 1.02 ^l^	144.52 ± 1.21 ^o^	152.65 ± 2.08 ^n^	262.77 ± 5.04 ^m^	262.75 ± 1.48 ^m^	1.35 ± 0.04 ^efg^	1.26 ± 0.03 ^hi^
1.5	4.7	67.27 ± 0.46 ^l^	119.72 ± 1.17 ^e^	142.93 ± 0.80 ^o^	270.26 ± 1.69 ^h^	257.20 ± 2.97 ^m^	478.94 ± 4.12 ^g^	1.33 ± 0.02 ^efg^	1.33 ± 0.00 ^efg^
1.5	5.6	80.11 ± 0.51 ^k^	104.10 ± 1.17 ^h^	174.34 ± 0.81 ^m^	239.45 ± 1.86 ^jk^	314.36 ± 4.36 ^l^	417.16 ± 2.60 ^j^	1.34 ± 0.03 ^efg^	1.31 ± 0.01 ^fgh^
Raw		22.46 ± 0.45 ^m^		46.32 ± 0.14 ^p^		93.11 ± 1.43 ^n^		1.53 ± 0.04 ^a^	

Different superscript letters within the same response variable indicate significant differences among treatment groups according to one-way ANOVA followed by Duncan’s multiple-range test (*p* < 0.05). P, atomization pressure; *F_nom_*, nominal binder pump setting. Raw SPI was included as a reference group for statistical comparison where applicable. During RH-controlled operation, effective binder delivery differed from *F_nom_*; the corresponding duty cycle and effective binder-delivery parameters are reported in Table 1.

Under continuous spray operation, increasing atomization pressure generally shifted the particle size distribution toward smaller diameters. At *F_nom_* = 3.8 mL·min^−1^, for example, *D*_50_ decreased from 336.31 µm at 0.5 bar to 234.98 µm at 1.0 bar and 144.52 µm at 1.5 bar. Similar decreases were observed at *F_nom_* = 4.7 and 5.6 mL·min^−1^. The corresponding decreases in *D*_10_ and *D*_90_ indicate that the entire distribution shifted toward smaller sizes as atomization pressure increased. Higher atomization pressure may produce smaller droplets and shorter liquid-bridge lifetimes, thereby restricting coalescence and granule growth [2,4,9]. However, possible contributions from particle breakage and attrition were not measured directly.

RH-controlled operation produced higher mean *D*_10_ and *D*_50_ values than continuous spraying in all nine operating conditions. The mean *D*_90_ value also increased in eight conditions and remained almost unchanged at 1.5 bar and 3.8 mL·min^−1^. For example, at 1.0 bar and *F_nom_* = 5.6 mL·min^−1^, RH-controlled operation increased *D*_10_ from 112.63 to 159.60 µm, *D*_50_ from 250.18 to 327.59 µm, and *D*_90_ from 453.07 to 564.24 µm.

The distribution span decreased under RH-controlled operation in seven of the nine conditions, indicating that the particle size distribution generally became narrower relative to its median diameter. At 1.0 bar and *F_nom_* = 5.6 mL·min^−1^, for example, span decreased from 1.36 to 1.24. However, span increased slightly from 1.47 to 1.52 at 1.0 bar and 3.8 mL·min^−1^ and remained unchanged at 1.5 bar and 4.7 mL·min^−1^. Therefore, the narrowing effect was common but not universal.

Overall, the RH-triggered strategy shifted the lower and central regions of the particle size distribution toward larger diameters and frequently reduced the relative distribution width. However, because *D*_90_ generally increased under RH control, the results do not support the conclusion that the strategy suppressed oversized particle growth. Furthermore, the controlled treatments received lower cumulative binder volumes and experienced intermittent spray sequences. The observed particle size changes should therefore be interpreted as combined responses to the regulated outlet-RH trajectory, effective binder delivery, and spray history rather than as an independent effect of RH regulation.

#### 3.4.2. Granule Morphology

**Figure 4:**
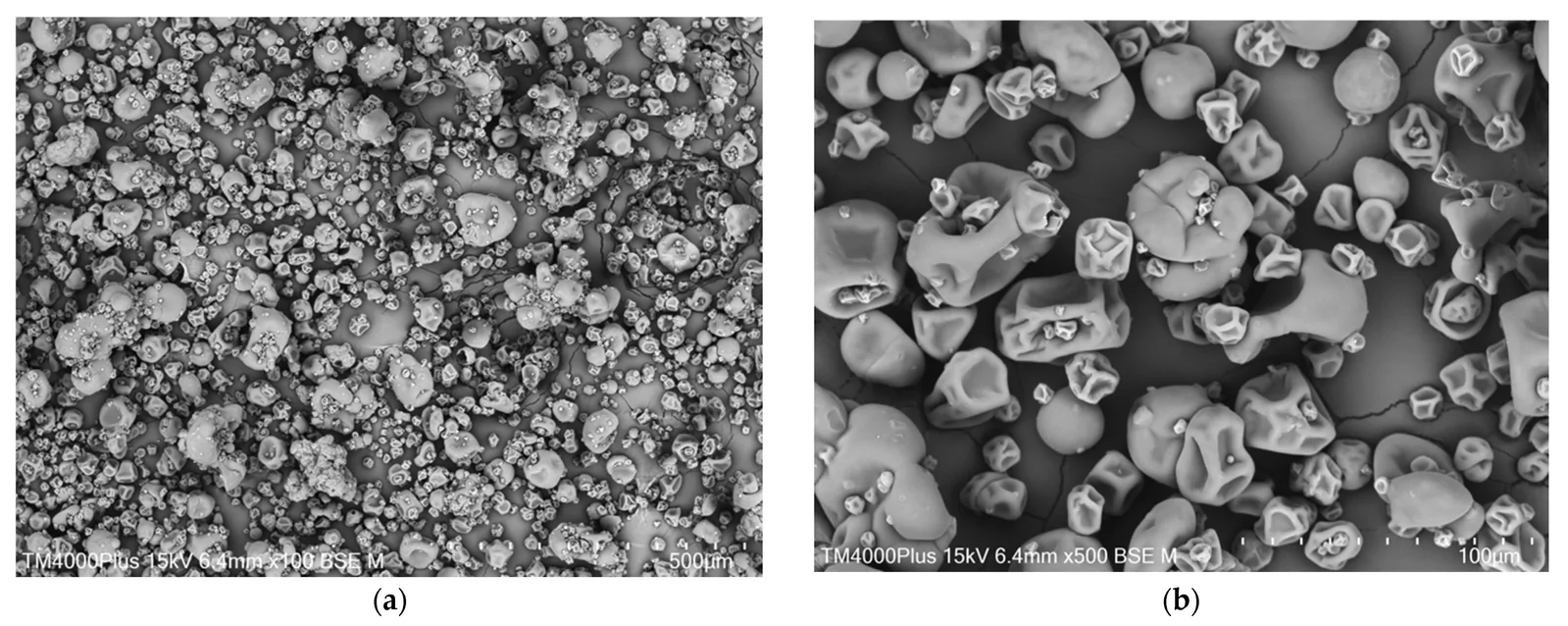
SEM micrographs of raw SPI powder at (**a**) ×100 and (**b**) ×500 magnification. Enlarged versions of all individual micrographs are provided in Supplementary Figure S1.

**Figure 5:**
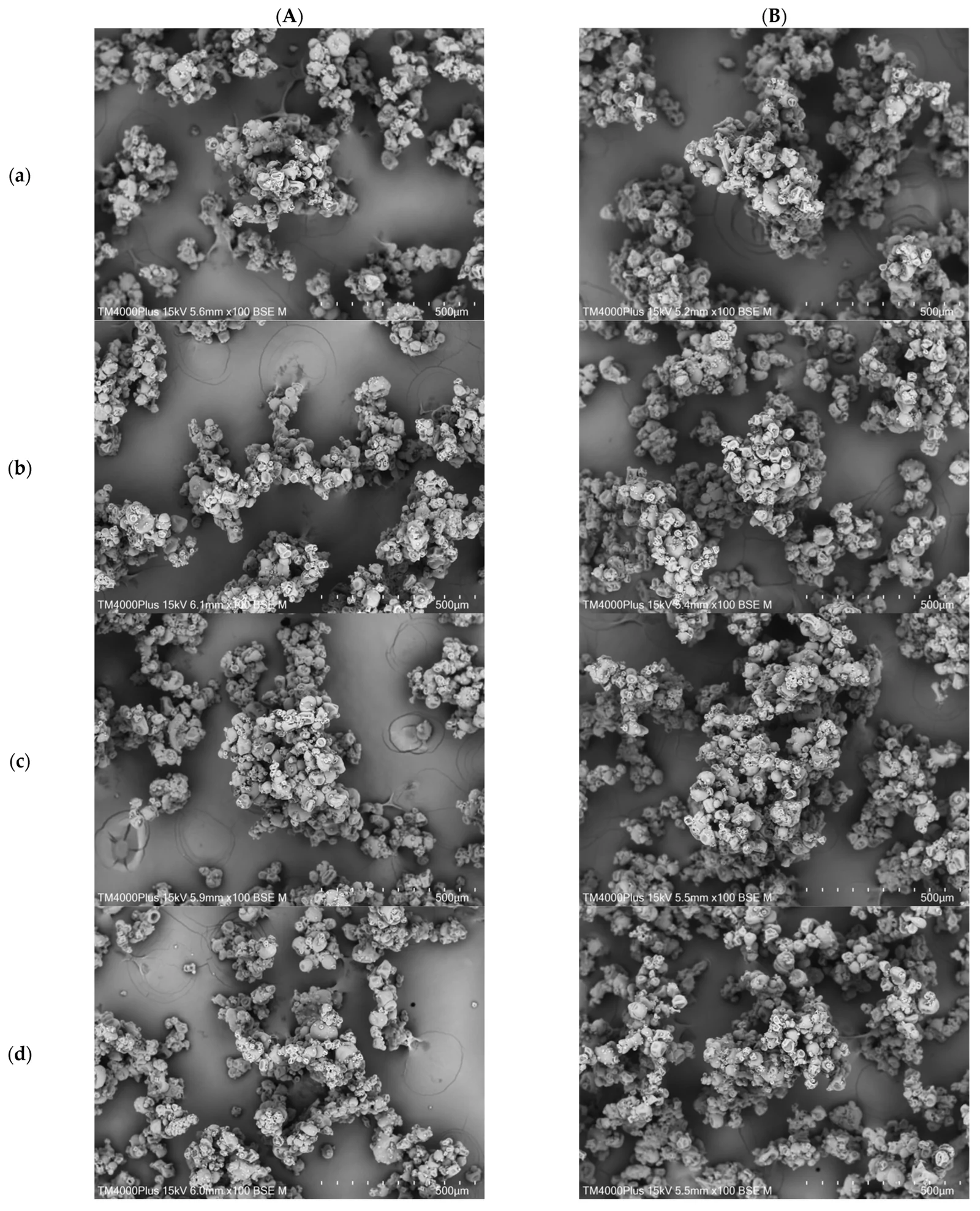

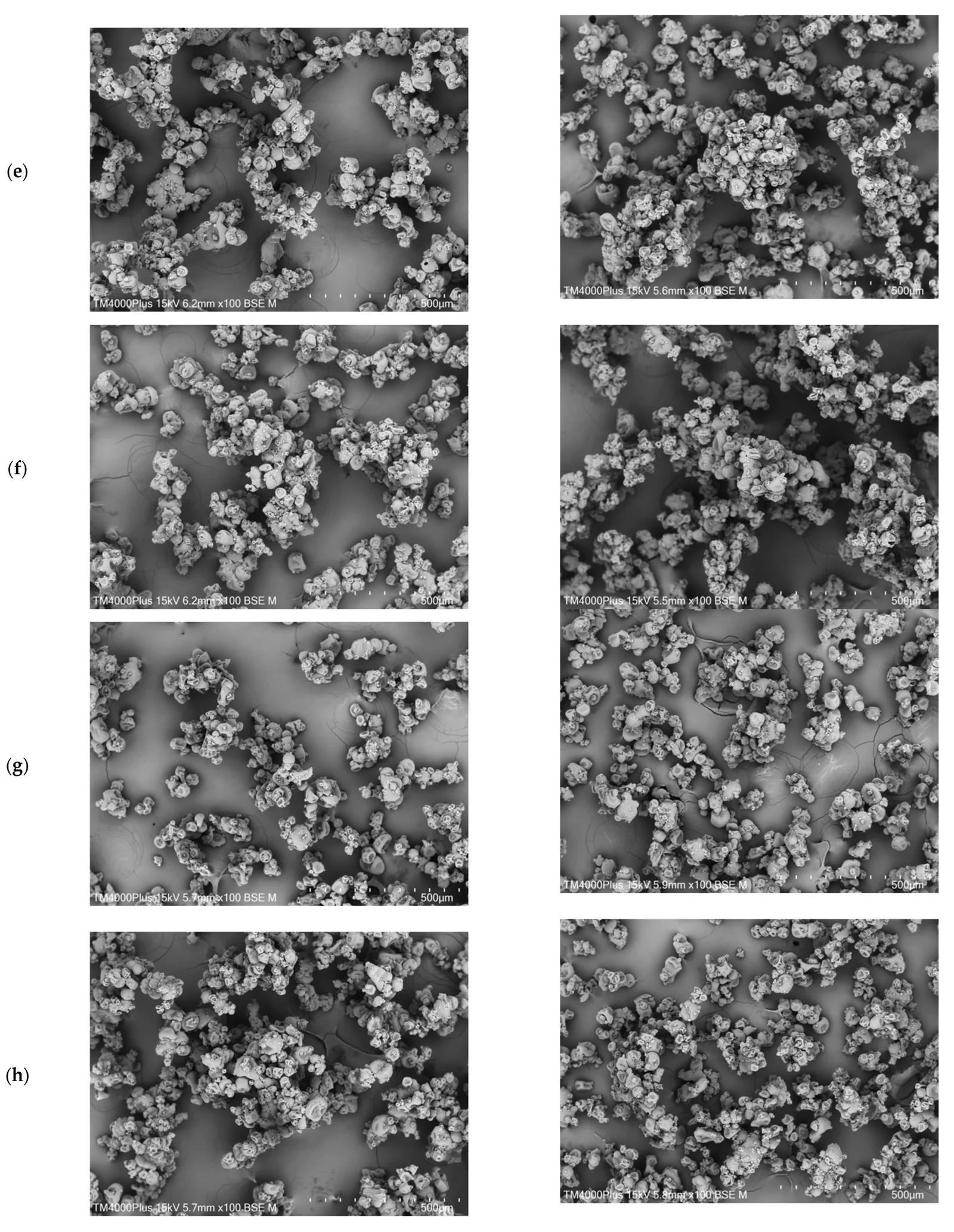

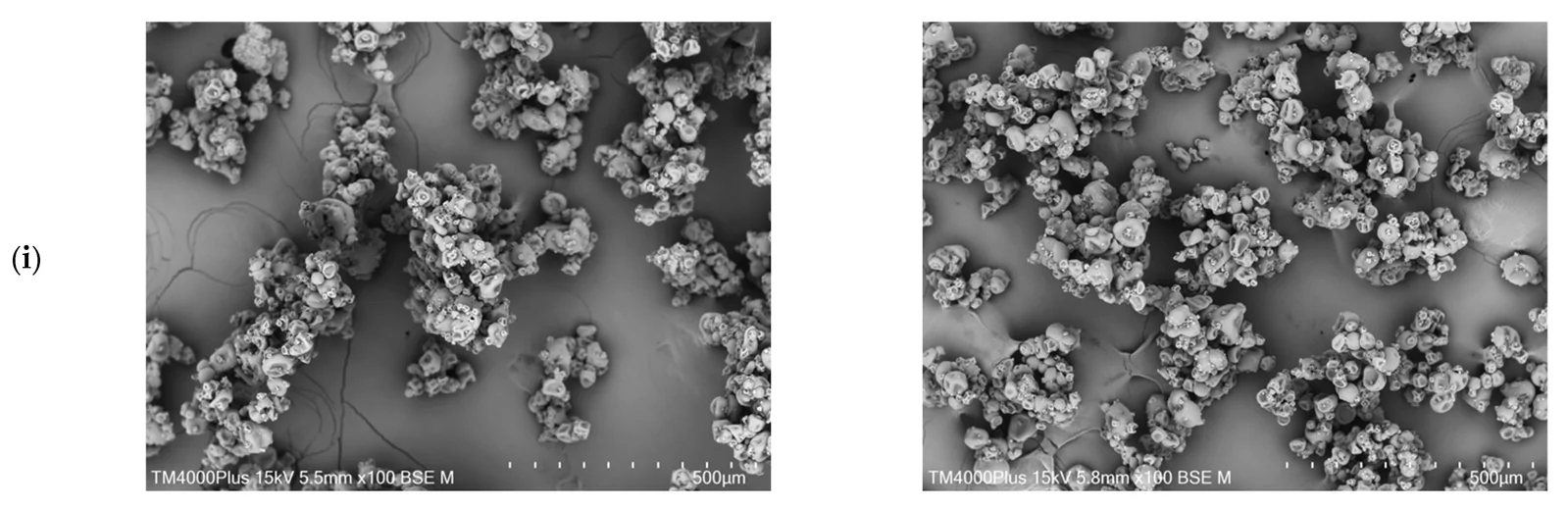
Representative SEM micrographs at ×100 magnification of SPI granules produced at (**a**) 0.5 bar and 3.8 mL·min^−1^, (**b**) 0.5 bar and 4.7 mL·min^−1^, (**c**) 0.5 bar and 5.6 mL·min^−1^, (**d**) 1.0 bar and 3.8 mL·min^−1^, (**e**) 1.0 bar and 4.7 mL·min^−1^, (**f**) 1.0 bar and 5.6 mL·min^−1^, (**g**) 1.5 bar and 3.8 mL·min^−1^, (**h**) 1.5 bar and 4.7 mL·min^−1^, and (**i**) 1.5 bar and 5.6 mL·min^−1^. Columns represent (**A**) continuous spray operation and (**B**) RH-controlled operation at a 70% set point. The images are representative two-dimensional fields acquired at ×100 magnification and are presented for qualitative morphological context only. No quantitative image analysis was performed. Enlarged versions of all individual micrographs are provided in Supplementary Figures S2–S10.

SEM micrographs were used only to provide qualitative morphological context for the quantitative particle size results. Raw SPI consisted of small particles with irregular shapes and heterogeneous surface features (Figure 4), consistent with its measured *D*_50_ and *D*_90_ values of 46.32 and 93.11 µm, respectively (Table 3). Following agglomeration, the samples qualitatively exhibited cluster-like structures formed through the attachment of smaller primary particles (Figure 5). Such structures are consistent with fluidized-bed agglomeration, in which wetted particles collide, form liquid bridges, and subsequently consolidate during drying [2,3,19].

Because the agglomerated sample micrographs represent selected two-dimensional fields acquired at a single magnification, visual differences among treatments were not used to establish treatment effects. Particle enlargement and changes in distribution width were evaluated primarily from the laser-diffraction results in Table 3. The SEM images cannot quantify fines content, internal granule porosity, granule strength, or morphological reproducibility. Reliable evaluation of these properties would require systematic field selection, replicate imaging of independent batches, multiple magnifications, quantitative image analysis, and, for internal porosity, cross sectional or three-dimensional characterization.

Because the SEM images represent selected two-dimensional fields of view, they provide qualitative rather than quantitative evidence of morphological differences. Therefore, the images cannot independently demonstrate reduced fines, improved reproducibility, greater granule strength, or increased internal porosity without additional image analysis or mechanical characterization.

### 3.5. Bulk Packing Properties

Table 4 summarizes the bulk density (*ρ_b_*), 50-tap apparent density (*ρ_t_*_,50_), particle density (*ρ_p_*), and apparent tapped-bed porosity (*ε_t_*) of the raw and agglomerated SPI powders. These properties provide information on powder-bed packing and the void space between particles [1,3,15,19].

**Table 4:** Bulk density, 50-tap apparent density, particle density, and apparent tapped-bed porosity of raw SPI and agglomerated products obtained at different atomization pressures (P) and nominal binder pump settings (*F_nom_*) under continuous spray and RH-controlled operation.

Condition		Bulk Density (g·mL^−1^)		50-Tap Apparent Density (g·mL^−1^)		Particle Density (g·mL^−1^)		Porosity (%)	
P	*F_nom_*	No Control	Control	No Control	Control	No Control	Control	No Control	Control
0.5	3.8	0.19 ± 0.01 ^i^	0.22 ± 0.01 ^fgh^	0.24 ± 0.00 ^gh^	0.27 ± 0.01 ^fgh^	1.22 ± 0.00 ^i^	1.26 ± 0.01 ^g^	80.34 ± 0.04 ^a^	78.69 ± 0.66 ^a^
0.5	4.7	0.19 ± 0.01 ^i^	0.22 ± 0.00 ^fgh^	0.25 ± 0.02 ^gh^	0.29 ± 0.01 ^def^	1.26 ± 0.00 ^gh^	1.31 ± 0.01 ^cd^	80.32 ± 1.48 ^a^	78.05 ± 0.61 ^abc^
0.5	5.6	0.21 ± 0.00 ^fghi^	0.23 ± 0.01 ^efgh^	0.26 ± 0.00 ^fgh^	0.28 ± 0.00 ^defg^	1.23 ± 0.01 ^i^	1.32 ± 0.01 ^c^	78.81 ± 0.22 ^a^	78.44 ± 0.08 ^ab^
1.0	3.8	0.25 ± 0.01 ^cde^	0.22 ± 0.01 ^fgh^	0.32 ± 0.01 ^cd^	0.28 ± 0.02 ^efg^	1.24 ± 0.00 ^hi^	1.30 ± 0.02 ^cde^	74.06 ± 0.93 ^d^	78.51 ± 1.60 ^ab^
1.0	4.7	0.20 ± 0.01 ^hi^	0.23 ± 0.00 ^def^	0.26 ± 0.01 ^fgh^	0.31 ± 0.02 ^cde^	1.22 ± 0.01 ^i^	1.29 ± 0.01 ^ef^	78.61 ± 1.05 ^ab^	75.88 ± 1.72 ^bcd^
1.0	5.6	0.22 ± 0.01 ^fgh^	0.21 ± 0.01 ^ghi^	0.29 ± 0.01 ^def^	0.26 ± 0.01 ^fgh^	1.32 ± 0.02 ^bc^	1.27 ± 0.01 ^fg^	78.24 ± 0.83 ^abc^	79.75 ± 0.97 ^a^
1.5	3.8	0.26 ± 0.02 ^bcd^	0.28 ± 0.01 ^b^	0.38 ± 0.02 ^b^	0.39 ± 0.00 ^b^	1.26 ± 0.01 ^g^	1.34 ± 0.01 ^ab^	70.18 ± 1.69 ^e^	70.61 ± 0.19 ^e^
1.5	4.7	0.23 ± 0.01 ^efg^	0.27 ± 0.03 ^bc^	0.31 ± 0.03 ^cde^	0.40 ± 0.02 ^b^	1.29 ± 0.02 ^ef^	1.29 ± 0.01 ^def^	75.73 ± 2.19 ^cd^	69.23 ± 1.45 ^e^
1.5	5.6	0.25 ± 0.02 ^bcd^	0.27 ± 0.02 ^b^	0.33 ± 0.04 ^c^	0.40 ± 0.04 ^b^	1.32 ± 0.01 ^cd^	1.35 ± 0.01 ^a^	74.80 ± 2.80 ^d^	70.27 ± 2.90 ^e^
Raw		0.30 ± 0.01 ^a^		0.55 ± 0.02 ^a^		1.22 ± 0.02 ^i^		54.73 ± 1.58 ^f^	

Different superscript letters within the same response variable indicate significant differences among treatment groups according to one-way ANOVA followed by Duncan’s multiple-range test (*p* < 0.05). P, atomization pressure; *F_nom_*, nominal binder pump setting. Raw SPI was included as a reference group for statistical comparison where applicable. During RH-controlled operation, effective binder delivery differed from *F_nom_*; the corresponding duty cycle and effective binder-delivery parameters are reported in Table 1.

Raw SPI exhibited a bulk density of 0.301 g·mL^−1^, a tapped density of 0.554 g·mL^−1^, and an apparent tapped-bed porosity of 54.73%. Following agglomeration, bulk density decreased to 0.191–0.256 g·mL^−1^ under continuous spray operation and 0.206–0.277 g·mL^−1^ under RH-controlled operation. Tapped density similarly decreased to 0.239–0.377 and 0.258–0.402 g·mL^−1^, respectively. Consequently, apparent tapped-bed porosity increased to 70.18–80.34% under continuous spraying and 69.23–79.75% under RH-controlled operation.

The lower bulk and tapped densities of the agglomerated powders relative to raw SPI indicate that particle enlargement produced more loosely packed powder beds. Fluidized-bed agglomeration commonly forms irregular clusters of attached primary particles, which occupy a greater bulk volume and create more interparticle void space than the original fine powder [1,2,3,15,19]. However, the apparent tapped-bed porosity calculated in this study describes the void fraction of the tapped powder bed and should not be interpreted as the internal porosity of individual granules.

Atomization pressure showed a relatively clear influence on the packing properties. Under continuous spray operation at *F_nom_* = 3.8 mL·min^−1^, increasing atomization pressure from 0.5 to 1.5 bar increased tapped density from 0.239 to 0.377 g·mL^−1^ and reduced apparent tapped-bed porosity from 80.34 to 70.18%. Similar trends were observed at nominal pump settings of 4.7 and 5.6 mL·min^−1^. These changes were consistent with the particle size results in Table 3, which showed a general shift toward smaller granules at higher atomization pressure. Particle size, shape, and agglomerate structure jointly influence the ability of a powder population to rearrange and pack during tapping [2,3,15,19].

Under RH-controlled operation, samples produced at 1.5 bar also generally exhibited higher bulk and tapped densities and lower apparent tapped-bed porosities than samples produced at 0.5 bar. Nevertheless, the response at 1.0 bar was not always intermediate, indicating that packing behavior was influenced by the combined effects of atomization pressure, effective binder delivery, particle size distribution, and granule morphology.

The effect of nominal binder pump setting was less systematic. Increasing liquid input may enhance particle coalescence and granule consolidation, but it may also produce larger or more irregular agglomerates that pack less efficiently. Therefore, changes in bulk and tapped densities with *F_nom_* should be interpreted together with the corresponding particle size and morphology results rather than as a simple monotonic response to binder input [2,3,4,7,19].

The effect of RH-controlled operation was also condition-dependent. At 0.5 bar, RH control increased bulk and tapped densities and slightly reduced apparent tapped-bed porosity at all three nominal pump settings. At 1.0 bar, however, RH control increased apparent tapped-bed porosity at *F_nom_* = 3.8 and 5.6 mL·min^−1^, but decreased it at 4.7 mL·min^−1^. These mixed responses indicate that the feedback strategy did not impose a uniform change in powder-bed packing. Because RH control also modified cumulative binder addition and spray timing, the observed differences likely resulted from the combined effects of outlet-RH history, wetting–drying sequence, and effective liquid input [6,7,8,9,13].

Particle density ranged from 1.217 to 1.325 g·mL^−1^ under continuous spray operation and from 1.261 to 1.351 g·mL^−1^ under RH-controlled operation. These variations did not follow a simple relationship with atomization pressure or nominal pump setting. Because pycnometric particle density and powder-bed packing density describe different physical properties, conclusions regarding bed packing should be based primarily on the combined trends in bulk density, tapped density, and apparent tapped-bed porosity [1,15,19].

### 3.6. Flowability and Cohesiveness

Table 5 presents the Carr compressibility index (*CI*) and Hausner ratio (*HR*) of the raw and agglomerated SPI powders. Both parameters are packing-derived indices calculated from bulk and tapped densities; lower values indicate better flowability and lower apparent cohesiveness [15,18]. Because *CI* and *HR* are derived from the same density measurements, they should be interpreted jointly rather than as independent indicators.

**Table 5:** Carr compressibility index (CI) and Hausner ratio (HR) of raw SPI and agglomerated products obtained at different atomization pressures (P) and nominal binder pump settings (*F_nom_*) under continuous spray and RH-controlled operation.

Condition		CI (%)		HR (-)	
P	*F_nom_*	No Control	Control	No Control	Control
0.5	3.8	20.32 ± 3.22 ^efg^	18.00 ± 0.00 ^g^	1.26 ± 0.05 ^efg^	1.22 ± 0.00 ^g^
0.5	4.7	22.73 ± 0.64 ^def^	23.64 ± 2.29 ^def^	1.29 ± 0.01 ^defg^	1.31 ± 0.04 ^def^
0.5	5.6	19.00 ± 0.00 ^fg^	20.53 ± 1.29 ^efg^	1.23 ± 0.00 ^fg^	1.26 ± 0.02 ^efg^
1.0	3.8	22.54 ± 1.28 ^defg^	20.63 ± 3.18 ^efg^	1.29 ± 0.02 ^defg^	1.26 ± 0.05 ^efg^
1.0	4.7	23.19 ± 1.92 ^def^	24.81 ± 5.09 ^de^	1.30 ± 0.03 ^defg^	1.33 ± 0.09 ^de^
1.0	5.6	24.73 ± 0.64 ^de^	20.27 ± 0.64 ^efg^	1.33 ± 0.01 ^de^	1.25 ± 0.01 ^efg^
1.5	3.8	32.00 ± 0.00 ^b^	29.79 ± 3.65 ^bc^	1.47 ± 0.00 ^b^	1.43 ± 0.08 ^bc^
1.5	4.7	26.34 ± 3.79 ^cd^	31.63 ± 3.83 ^b^	1.36 ± 0.07 ^cd^	1.47 ± 0.08 ^b^
1.5	5.6	22.89 ± 2.52 ^def^	31.45 ± 1.26 ^b^	1.30 ± 0.04 ^defg^	1.46 ± 0.03 ^b^
Raw		45.73 ± 0.64 ^a^		1.84 ± 0.02 ^a^	

Different superscript letters within the same response variable indicate significant differences among treatment groups according to one-way ANOVA followed by Duncan’s multiple-range test (*p* < 0.05). P, atomization pressure; *F_nom_*, nominal binder pump setting; CI, Carr compressibility index; HR, Hausner ratio. Raw SPI was included as a reference group for statistical comparison where applicable. During RH-controlled operation, effective binder delivery differed from *F_nom_*; the corresponding duty cycle and effective binder-delivery parameters are reported in Table 1. CI and HR were calculated using *ρ*_t,50_ and represent packing-derived screening indices rather than direct measurements of dynamic powder flow.

Raw SPI exhibited a *CI* of 45.73% and an HR of 1.84, corresponding to very poor flowability and high apparent cohesiveness. Following agglomeration, *CI* decreased to 18.00–32.00%, while *HR* decreased to 1.22–1.47. Thus, the agglomerated products exhibited good-to-fair flowability and intermediate-to-high apparent cohesiveness according to the classification described in Section 2.4.4. The marked improvement relative to raw SPI was consistent with particle enlargement and agglomerate formation, which reduce the relative influence of cohesive interparticle forces and alter powder-bed packing behavior [2,3,15,19].

Under continuous spray operation, increasing atomization pressure generally increased *CI* and *HR*, although the effect depended on the nominal binder pump setting. At *F_nom_* = 3.8 mL·min^−1^, *CI* increased from 20.32% at 0.5 bar to 22.54% at 1.0 bar and 32.00% at 1.5 bar, while *HR* increased from 1.26 to 1.29 and 1.47, respectively. A similar, although smaller, deterioration was observed at F_nom_ = 4.7 mL·min^−1^. These trends were consistent with the shift toward smaller particle sizes at higher atomization pressure shown in Table 3. Finer particle populations generally exhibit poorer flow because cohesive surface forces become more influential relative to particle weight [2,3,15,19].

The effect of the nominal binder pump setting was not monotonic. Under continuous spraying at 1.5 bar, increasing *F_nom_* from 3.8 to 5.6 mL·min^−1^ reduced *CI* from 32.00 to 22.89% and *HR* from 1.47 to 1.30. In contrast, at 1.0 bar, both indices increased with the nominal pump setting. Increasing liquid input may promote particle growth and reduce the influence of fine particles, but excessive or non-uniform wetting may also increase liquid-bridge formation, powder compressibility, and cohesiveness [2,3,6,7,8,19]. Consequently, flow behavior depended on the combined effects of particle size distribution, moisture history, morphology, and powder-bed packing.

RH-controlled operation produced condition-dependent changes in *CI* and *HR*. The mean *CI* and *HR* values decreased in four of the nine controlled–uncontrolled comparisons and increased in five. For example, at 1.0 bar and *F_nom_* = 5.6 mL·min^−1^, RH control reduced *CI* from 24.73 to 20.27% and *HR* from 1.33 to 1.25. Conversely, at 1.5 bar and *F_nom_* = 5.6 mL·min^−1^, *CI* increased from 22.89 to 31.45% and *HR* increased from 1.30 to 1.46. At 1.5 bar, the increases observed at nominal pump settings of 4.7 and 5.6 mL·min^−1^ were statistically significant according to the superscript comparisons in Table 5.

These mixed responses demonstrate that RH-triggered operation did not uniformly improve flowability. In particular, the larger median particle sizes observed under several controlled conditions did not always produce lower *CI* and *HR*, confirming that particle size alone does not determine powder flow; particle shape, size distribution, surface characteristics, and packing behavior also contribute [2,3,15,19]. Moreover, RH-controlled treatments differed from continuous spray treatments in cumulative binder delivery and spray sequence. Differences in *CI* and *HR* should therefore be attributed to the complete RH-triggered binder-delivery strategy rather than to RH regulation alone [6,7,8,9,13].

Overall, agglomeration decreased *CI* and *HR* relative to raw SPI, indicating improvement in the packing-derived flow indices obtained using the fixed 50-tap procedure. However, the effect of RH-triggered binder diversion depended on atomization pressure and effective binder delivery. Because an equilibrium tapped volume was not confirmed and dynamic flow was not measured, *CI* and *HR* should be interpreted together with particle size, morphology, and powder-packing results rather than as direct evidence of flow performance in processing equipment [15,18,19].

### 3.7. Reconstitution Properties

Reconstitution involves several partially overlapping stages, including wetting and sinking, dispersion through agglomerate disintegration, and dissolution or solubilization of the primary particles. Improvement in one stage does not necessarily result in improvement in the others [1,26]. Table 6 presents the wetting time and dispersibility of the raw SPI and agglomerated products.

**Table 6:** Wetting time and dispersibility of raw SPI and agglomerated products obtained at different atomization pressures (P) and nominal binder pump settings (*F_nom_*) under continuous spray and RH-controlled operation.

Condition		Wetting Time (s)		Dispersibility (%)	
P	*F_nom_*	No Control	Control	No Control	Control
0.5	3.8	43.72 ± 2.60 ^bc^	26.91 ± 0.87 ^e^	41.43 ± 1.99 ^i^	46.08 ± 9.01 ^ghi^
0.5	4.7	44.08 ± 8.66 ^bc^	34.82 ± 1.73 ^bcde^	54.00 ± 7.32 ^efg^	51.50 ± 6.98 ^fgh^
0.5	5.6	29.54 ± 4.33 ^de^	42.08 ± 8.66 ^bc^	37.71 ± 3.71 ^ij^	46.27 ± 0.53 ^ghi^
1.0	3.8	43.96 ± 8.11 ^bc^	27.82 ± 1.73 ^e^	43.37 ± 7.67 ^hi^	43.41 ± 1.43 ^hi^
1.0	4.7	34.91 ± 0.87 ^bcde^	40.17 ± 7.79 ^bcd^	53.66 ± 6.74 ^efg^	58.04 ± 4.86 ^cdef^
1.0	5.6	33.08 ± 8.66 ^cde^	35.72 ± 2.60 ^bcde^	30.41 ± 0.85 ^j^	41.06 ± 4.25 ^i^
1.5	3.8	39.36 ± 6.06 ^bcd^	45.05 ± 7.29 ^b^	69.24 ± 8.88 ^b^	62.19 ± 3.34 ^bcde^
1.5	4.7	36.23 ± 5.69 ^bcde^	37.27 ± 6.93 ^bcde^	65.40 ± 5.43 ^bcd^	67.33 ± 0.61 ^bc^
1.5	5.6	40.96 ± 8.11 ^bcd^	33.63 ± 3.46 ^bcde^	57.28 ± 6.82 ^def^	60.28 ± 1.43 ^bcdef^
Raw		83.54 ± 4.33 ^a^		95.80 ± 4.41 ^a^	

Different superscript letters within the same response variable indicate significant differences among treatment groups according to one-way ANOVA followed by Duncan’s multiple-range test (*p* < 0.05). P, atomization pressure; *F_nom_*, nominal binder pump setting. During RH-controlled operation, effective binder delivery differed from *F_nom_*; the corresponding duty cycle and effective binder-delivery parameters are reported in Table 1.

Raw SPI exhibited a wetting time of 83.54 s and a dispersibility of 95.80%. Following agglomeration, wetting time decreased to 26.91–45.05 s, whereas dispersibility decreased to 30.41–69.24%. These opposing changes demonstrate that wetting and dispersion represent different stages of reconstitution. Wetting describes the initial displacement of air from the powder surface and the penetration and sinking of the powder into water, whereas dispersion requires subsequent disintegration of the wetted agglomerates and release of the primary particles into the surrounding liquid [1,26,27]. Improvement in the first stage therefore does not necessarily ensure improvement in the second.

The shorter wetting times were consistent with the particle enlargement measured by laser diffraction and the cluster-like structures qualitatively observed by SEM. The median particle diameter increased from 46.32 µm for raw SPI to 142.93–384.65 µm for the agglomerated powders, while the SEM images showed clusters formed through the attachment of smaller primary particles. Larger agglomerates reduce the relative influence of cohesive forces that promote the floating of fine particles. In addition, spaces between the constituent particles can provide pathways for capillary water penetration, allowing water to enter an agglomerate from several contact points [18,26,27]. However, because internal pore size and porosity of individual agglomerates were not measured, capillary penetration should be regarded as a mechanism consistent with the observed particle enlargement and morphology rather than as a directly demonstrated structural property.

Dispersion requires additional structural changes after initial wetting. During granulation, water forms liquid bridges between adjacent SPI particles. Evaporation during subsequent drying can consolidate these contact regions into solid bridges. During reconstitution, water must penetrate the contact regions and the bridges must dissolve, erode, or rupture before the agglomerates can separate into primary particles [2,3,26,27]. Consequently, an agglomerate may become wetted rapidly while remaining mechanically intact during a short mixing period. Stronger or more numerous interparticle bridges would therefore favor agglomerate survival during the dispersion test, even if the external surface had already become completely wetted.

The particle size distribution and the dispersibility method provide additional explanation for the observed decrease. Dispersibility was determined after only 15 s of mixing, comprising 25 back-and-forth movements, followed by passage through a 212 µm sieve. The *D*_90_ values of all agglomerated powders ranged from 257.20 to 666.09 µm and therefore exceeded the sieve aperture, whereas the *D*_90_ of raw SPI was only 93.11 µm. Intact or partially disintegrated agglomerates would consequently be retained on the sieve, reducing the measured dispersibility. Thus, the lower values primarily indicate incomplete or slow agglomerate breakup under the selected mixing conditions. They should not be interpreted directly as evidence of reduced molecular protein solubility.

The effects of atomization pressure and nominal binder pump setting were not monotonic. Under continuous spraying at *F_nom_* = 4.7 mL·min^−1^, increasing atomization pressure from 0.5 to 1.5 bar reduced wetting time from 44.08 to 36.23 s. In contrast, at *F_nom_* = 5.6 mL·min^−1^, wetting time increased from 29.54 to 40.96 s over the same pressure range. Furthermore, the continuous spray treatment at 0.5 bar and *F_nom_* = 5.6 mL·min^−1^ exhibited the shortest wetting time among the uncontrolled treatments but relatively low dispersibility. Conversely, the treatment at 1.5 bar and *F_nom_* = 3.8 mL·min^−1^ had a longer wetting time but the highest dispersibility among the agglomerated samples. These results confirm that particle size alone did not determine reconstitution behavior. Agglomerate architecture, surface accessibility, bridge strength, and wetting–drying history were also likely to contribute [1,18,26,27].

RH-controlled operation produced condition-dependent changes in both responses. The mean wetting time decreased in four of the nine controlled–uncontrolled comparisons and increased in five. The clearest reductions occurred at *F_nom_* = 3.8 mL·min^−1^, where RH-triggered operation reduced wetting time from 43.72 to 26.91 s at 0.5 bar and from 43.96 to 27.82 s at 1.0 bar. Conversely, at 0.5 bar and *F_nom_* = 5.6 mL·min^−1^, wetting time increased from 29.54 to 42.08 s. Mean dispersibility increased under RH-controlled operation in seven of the nine conditions, although most controlled–uncontrolled differences were not statistically significant.

Interestingly, RH-controlled operation produced higher *D*_50_ values in all nine conditions, while its mean dispersibility was also higher in seven conditions. This apparent decoupling further indicates that agglomerate size alone did not control dispersion. The lower cumulative binder delivery under RH-triggered operation may have reduced the number or strength of consolidated interparticle bridges, thereby facilitating agglomerate breakup. Conversely, the intermittent wetting and drying intervals may have altered bridge consolidation, surface structure, and the spatial distribution of particle contacts [7,13,26,28]. Because granule mechanical strength and internal pore structure were not measured, the relative contributions of these mechanisms cannot be distinguished from the present results. Furthermore, the controlled treatments differed in cumulative binder volume and spray sequence; therefore, the reconstitution responses should be attributed to the complete RH-triggered binder-delivery strategy rather than to RH regulation alone.

Possible changes in SPI structure during thermal processing cannot be excluded because heat treatment can alter protein conformation, surface hydrophobicity, and protein–protein interactions [29]. Nevertheless, inlet-air temperature does not represent the actual product temperature, and neither product temperature, protein denaturation, nor protein solubility was measured. The reduction in dispersibility therefore cannot be attributed specifically to heat-induced protein denaturation or aggregation.

Although agglomeration shortened wetting time, the reduction in dispersibility from 95.80% for raw SPI to 30.41–69.24% may restrict its practical use in instant products requiring rapid and complete reconstitution. The present measurements cannot determine whether this reduction resulted from stronger interparticle bridges, heat-induced protein changes, insufficient agglomerate disintegration, or a combination of these mechanisms. Future studies should therefore evaluate agglomerate strength and disintegration kinetics, product-temperature history, protein denaturation, and protein solubility.

### 3.8. Study Limitations

This feasibility study included only two independent agglomeration runs per treatment. Although analytical replicates were averaged within each run to avoid pseudoreplication, the resulting standard deviations and statistical comparisons provide limited estimates of between-run variability and should be interpreted cautiously. Moreover, the RH descriptors were derived from one displayed trajectory per condition and therefore do not establish run-to-run reproducibility of the control dynamics. Outlet-air RH was an indirect, temperature-dependent gas-phase signal, while outlet-air temperature, absolute humidity, and real-time bed or powder moisture were not measured. Bed moisture, pressure drop, wall deposition, and fluidization stability were not monitored. Therefore, the outlet-RH trajectories cannot confirm the occurrence of overwetting or bed instability or demonstrate that these phenomena were prevented by the feedback controller. Process yield was calculated on a wet-mass basis using only the recovered chamber product passing through the 850 µm sieve; other material streams were not quantified, so the reported value does not constitute a full material balance. Finally, because feedback control altered both cumulative binder addition and spray history, the product differences cannot be attributed to RH regulation alone or extrapolated directly to industrial performance. Future studies should include additional independent batches, matched controls, direct moisture measurements, and a component-level material balance.

## 4. Conclusions

Outlet-air RH-triggered binder diversion was successfully implemented during pulsed fluidized-bed agglomeration of soy protein isolate. Under continuous spray operation, outlet RH showed an overall upward trend throughout the 30 min process. In contrast, RH-controlled operation produced a regulated outlet-RH regime near the selected 70% set point after threshold attainment. The controller adjusted the spray duty cycle from 0.67 to 0.96, corresponding to approximately 4–33% less cumulative binder delivery than continuous spraying at the same nominal pump setting. These findings demonstrate regulation of the measured outlet-air RH but do not establish that the controller prevented powder overwetting, excessive granule growth, wall deposition, or unstable fluidization.

Relative to continuous spray operation, RH-controlled operation produced a lower mean final moisture content in all nine conditions, a higher mean process yield in seven conditions, higher mean *D*_10_ and *D*_50_ values in all conditions, and a lower mean particle size span in seven conditions. Among the tested conditions, RH-controlled operation at an atomization pressure of 0.5 bar and a nominal binder pump setting of 4.7 mL·min^−1^ produced the highest mean process yield (74.22 ± 3.66%), together with a *D*_50_ of 359.22 ± 5.43 µm. However, this yield was not significantly different from several other high-yield treatments, and no single condition provided the most favorable values for all powder-quality responses. This condition should therefore be regarded as producing the highest observed mean yield within the tested range rather than as a definitive optimum. Pulsed fluidized-bed agglomeration reduced *CI* and *HR* and shortened wetting time compared with raw SPI. However, the effects of RH-controlled operation on powder-bed packing, Carr index, Hausner ratio, wetting time, and dispersibility depended on atomization pressure and effective binder delivery. Although agglomeration shortened wetting time, it reduced dispersibility relative to raw SPI. The substantial reduction in dispersibility remains an important practical limitation that requires further mechanistic investigation and process optimization.

Because the feedback system regulated outlet RH by intermittently diverting the binder stream, controlled treatments differed from continuous spray treatments in cumulative liquid addition and temporal spray sequence. Therefore, the observed product responses represent the performance of the complete RH-triggered adaptive binder-delivery strategy rather than an independent effect of RH stabilization alone. Because only one engineering demonstration set point was evaluated, no optimum or critical outlet-RH threshold for SPI agglomeration can be inferred from the present results. Future studies should include matched-binder-volume and matched spray sequence controls, evaluate additional RH set points, and monitor outlet-air temperature or absolute humidity to identify the independent contribution and optimum operating range of RH feedback control.

## Data Availability

The original contributions presented in this study are included in the article/Supplementary Materials. Further inquiries can be directed to the corresponding author.

## Supplementary Materials

The following supporting information can be downloaded at: https://www.mdpi.com/article/10.3390/foods15162846/s1, Table S1, Quantitative descriptors of outlet-air relative-humidity dynamics during continuous spray and RH-controlled agglomeration, and Figures S1–S10, Enlarged SEM micrographs corresponding to Figure 4 and Figure 5.
