# Supplementary material for: Outlet-Air Relative-Humidity Feedback for Adaptive Binder Delivery During Pulsed Fluidized-Bed Agglomeration of Soy Protein Isolate
Source: Foods. 2026 Aug 14;15(16):2846. doi: 10.3390/foods15162846 (PMC13512069; doi:10.3390/foods15162846)
Supplement: Supplementary file 1 [file foods-15-02846-s001.zip › foods-4438328-supplementary.pdf]

Table S1. Quantitative descriptors of outlet-air relative-humidity dynamics during continuous-spray and RH-controlled agglomeration.

Mode	P (bar)	F_{nom} (mL·min^{-1})	RH$_0$ (%)	RH$_f$ (%)	$r_{0\text{-}300}$ (%RH·s^{-1})	$r_{300\text{-}900}$ (%RH·s^{-1})	$r_{1200\text{-}1800}$ (%RH·s^{-1})	t_{70} (s)	$T_{>70}$ (min)	AUC$_{>70}$ (%RH·min)
CS	0.5	3.8	27.1	73.7	0.031	0.042	0.014	1512	4.77	11.1
CS	0.5	4.7	28.7	74.7	0.059	0.035	0.011	1279	8.67	26.4
CS	0.5	5.6	29.6	79.9	0.064	0.029	0.012	1041	12.60	72.1
CS	1.0	3.8	27.9	72.9	0.043	0.029	0.010	1446	5.90	12.2
CS	1.0	4.7	22.9	76.7	0.065	0.041	0.012	1227	9.38	39.5
CS	1.0	5.6	35.6	80.6	0.056	0.023	0.014	1065	12.25	77.8
CS	1.5	3.8	31.5	73.8	0.023	0.031	0.013	1435	6.08	13.2
CS	1.5	4.7	29.1	76.2	0.035	0.033	0.015	1307	8.23	28.4
CS	1.5	5.6	20.5	85.8	0.079	0.036	0.014	952	14.15	141.4
RHC	0.5	3.8	25.6	69.8	0.022	0.041	0.007	1469	1.13	0.2
RHC	0.5	4.7	28.7	70.3	0.036	0.035	0.003	1338	6.40	2.6
RHC	0.5	5.6	38.3	69.8	0.056	0.027	0.000	1357	6.30	1.6
RHC	1.0	3.8	25.8	70.2	0.055	0.041	0.006	1441	2.13	0.9
RHC	1.0	4.7	36.7	69.3	0.014	0.034	-0.003	1180	6.05	4.5
RHC	1.0	5.6	31.1	68.9	0.074	0.026	0.000	1170	3.05	1.2
RHC	1.5	3.8	29.0	70.1	0.027	0.029	0.010	1552	2.73	0.5
RHC	1.5	4.7	28.7	70.0	0.035	0.035	0.002	1321	4.95	1.4

RHC	1.5	5.6	31.1	69.9	0.044	0.031	0.000	966	6.63	3.0

Values are descriptive single-run metrics calculated from the individual outlet-air RH profiles shown in Figures 2 and 3; they are not replicate means. RH_0 and RH_f are the mean RH values during the first 30 s and final 60 s, respectively. The terms $r_{0–300}$, $r_{300–900}$, and $r_{1200–1800}$ are RH change rates estimated over 0–300, 300–900, and 1200–1800 s, respectively. The term t_{70} is the first time at which the 30-s moving-average RH reached the measured 70% controller set point. $T_{>70}$ is the cumulative time for which measured RH exceeded this set point, and $AUC_{>70}$ is the corresponding integrated RH excess, expressed as %RH·min. CS and RHC denote continuous-spray and RH-controlled operation, respectively. RH values represent measured sensor outputs. Because the stated sensor accuracy was ±2% RH, small differences within this accuracy range were not interpreted as physically meaningful. $T_{>70}$ and $AUC_{>70}$ should therefore be interpreted as descriptive metrics referenced to the measured controller set point rather than as precise exposure above an absolute true-RH threshold.

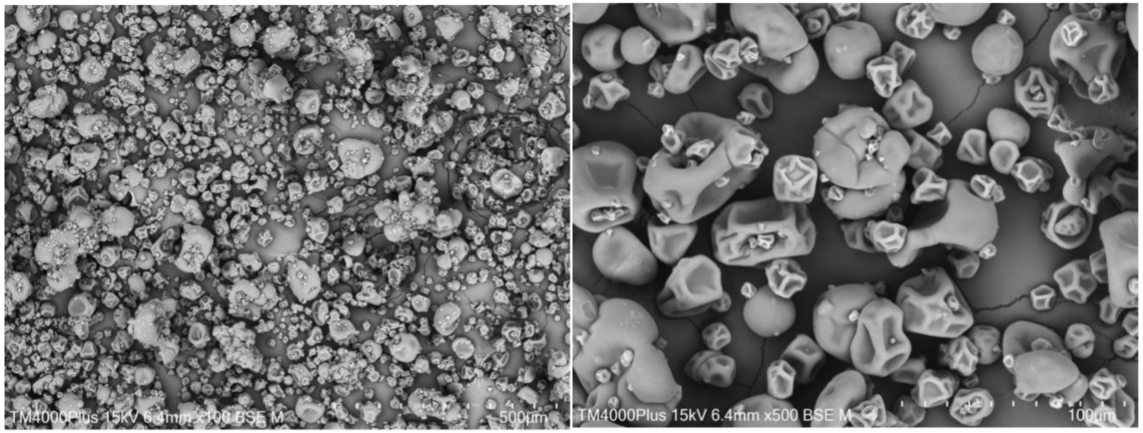

Figure S1. Enlarged SEM micrographs corresponding to Figure 4.

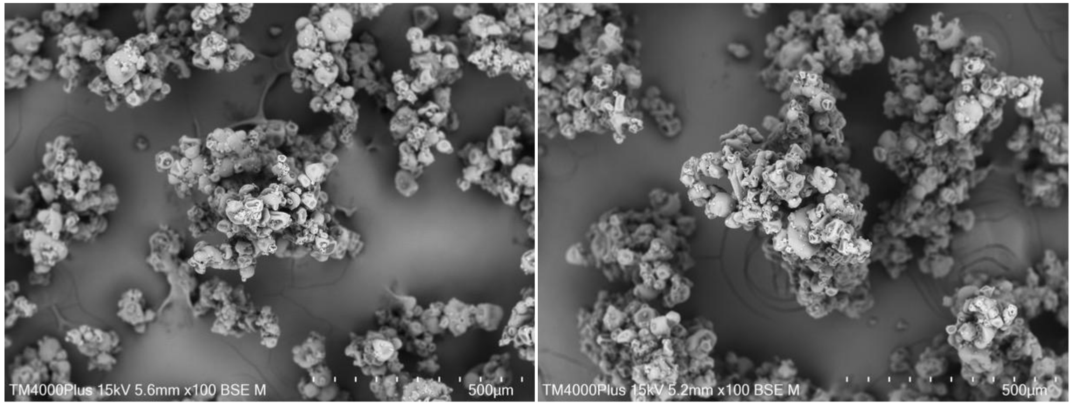

Figure S2. Enlarged SEM micrographs corresponding to Figure 5a.

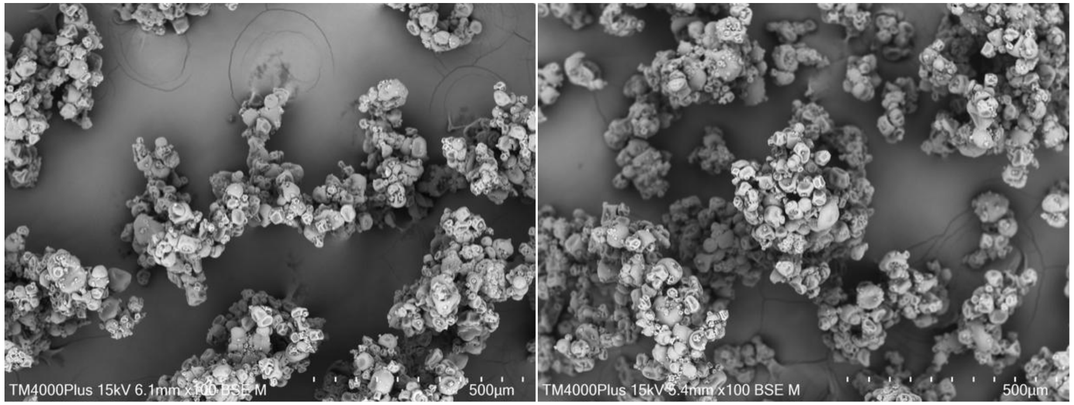

Figure S3. Enlarged SEM micrographs corresponding to Figure 5b.

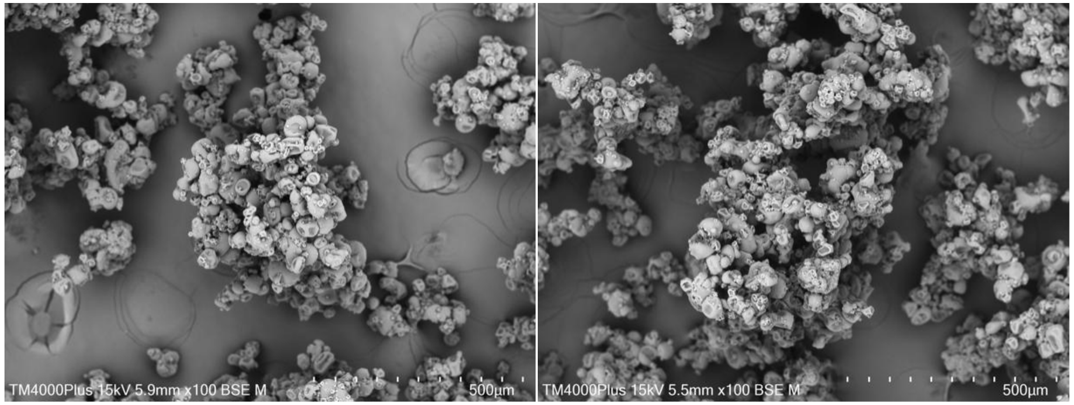

Figure S4. Enlarged SEM micrographs corresponding to Figure 5c.

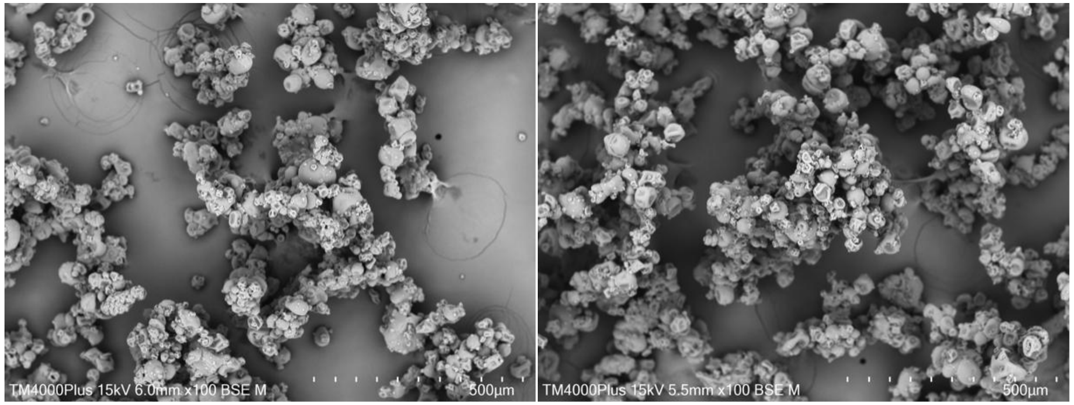

Figure S5. Enlarged SEM micrographs corresponding to Figure 5d.

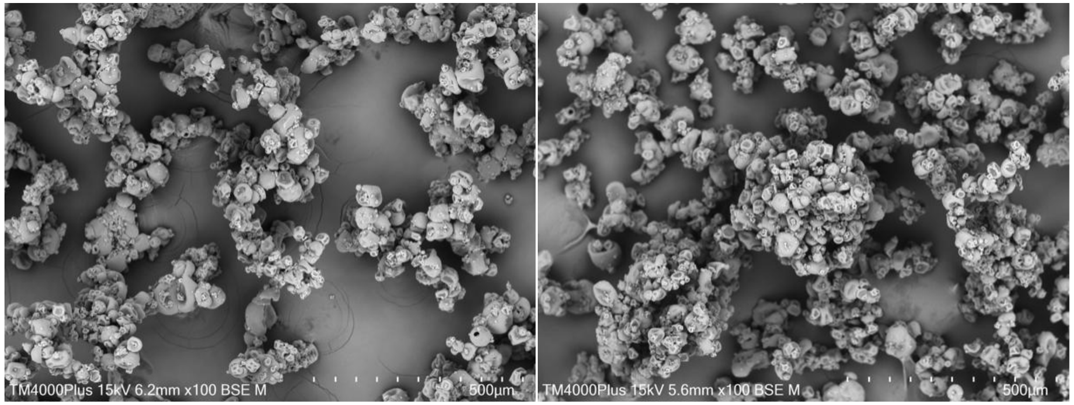

Figure S6. Enlarged SEM micrographs corresponding to Figure 5e.

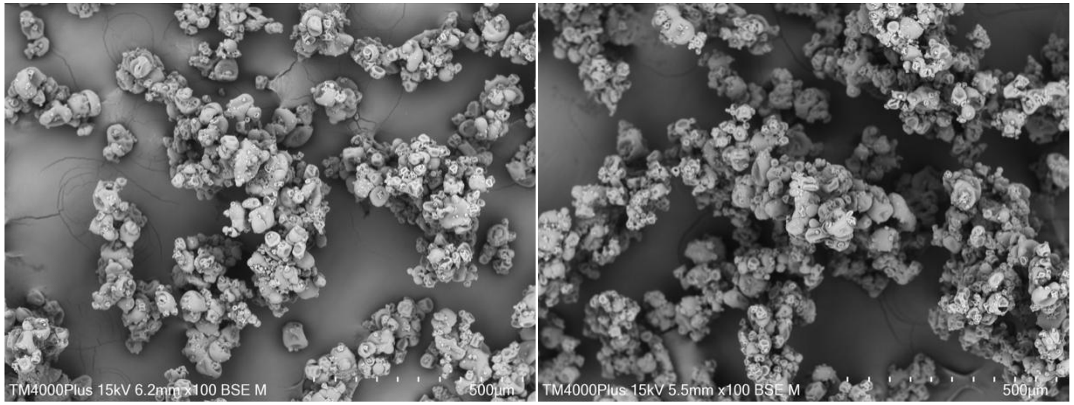

Figure S7. Enlarged SEM micrographs corresponding to Figure 5f.

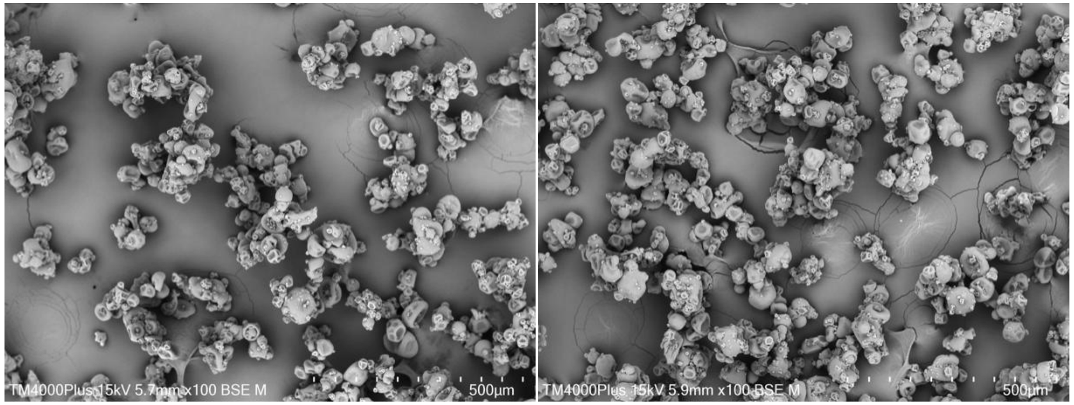

Figure S8. Enlarged SEM micrographs corresponding to Figure 5g.

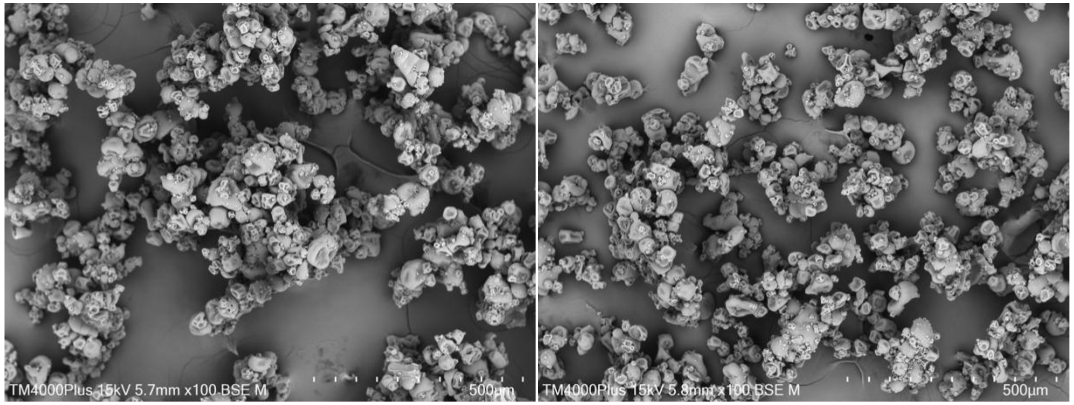

Figure S9. Enlarged SEM micrographs corresponding to Figure 5h.

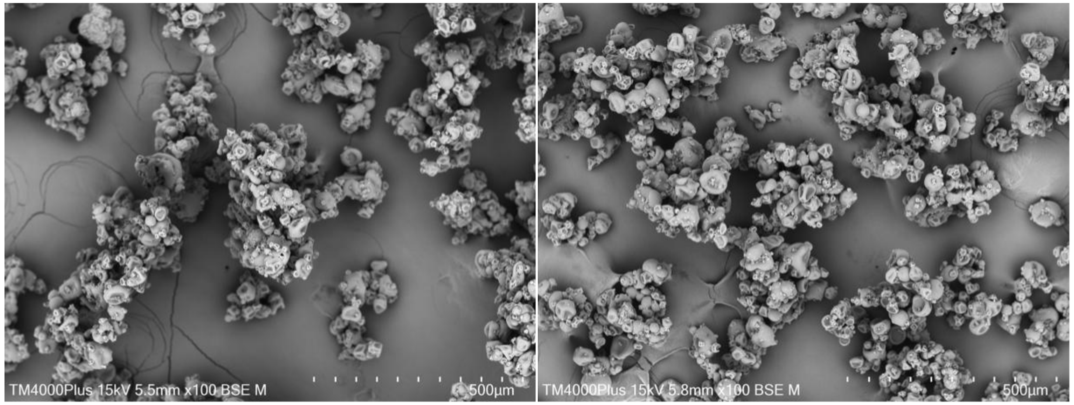

Figure S10. Enlarged SEM micrographs corresponding to Figure 5i.
